# The impact of the COVID-19 pandemic on rates and predictors of missed hospital appointments in multiple outpatient clinics of The Royal Hospital, Sultanate of Oman: a retrospective study

**DOI:** 10.1186/s12913-023-10395-w

**Published:** 2023-12-19

**Authors:** Ahmed Alawadhi, Victoria Palin, Tjeerd van Staa

**Affiliations:** 1 Centre for Health Informatics, School of Health Sciences, Faculty of Biology, Medicine and Health, The University of Manchester, Oxford Road, Manchester, M13 9PL UK; 2 Maternal and Fetal Research Centre, Division of Developmental Biology and Medicine, The Univeristy of Manchester, St Marys Hospital, Oxford Road, Manchester, M13 9WL UK

**Keywords:** Missed appointments, Impact of COVID-19, Virtual clinics, Oman

## Abstract

**Background:**

The global outbreak of the COVID-19 pandemic resulted in significant changes in the delivery of health care services such as attendance of scheduled outpatient hospital appointments. This study aimed to evaluate the impact of COVID-19 on the rate and predictors of missed hospital appointment in the Sultanate of Oman.

**Methods:**

A retrospective single-centre analysis was conducted to determine the effect of COVID-19 on missed hospital appointments at various clinics at The Royal Hospital (tertiary referral hospital) in Muscat, Sultanate of Oman. The study population included scheduled face-to-face and virtual appointments between January 2019 and March 2021. Logistic regression models were used with interaction terms (post COVID-19) to assess changes in the predictors of missed appointments.

**Results:**

A total of 34, 3149 scheduled appointments was analysed (320,049 face-to-face and 23,100 virtual). The rate of missed face-to-face hospital appointments increased from 16.9% pre to 23.8% post start of COVID-19, particularly in early pandemic (40.5%). Missed hospital appointments were more frequent (32.2%) in virtual clinics (post COVID-19). Increases in missed face-to-face appointments varied by clinic (Paediatrics from 19.3% pre to 28.2% post; Surgery from 12.5% to 25.5%; Obstetrics & Gynaecology from 8.4% to 8.5%). A surge in the frequency of missed appointments was seen during national lockdowns for face-to-face and virtual appointments. Most predictors of missed appointments did not demonstrate any appreciable changes in effect (i.e., interaction term not statistically significant). Distance of patient residence to the hospital revealed no discernible changes in the relative effect pre and post COVID-19 for both face-to-face and virtual clinic appointments.

**Conclusion:**

The rate of missed visits in most clinics was directly impacted by COVID-19. The case mix of patients who missed their appointments did not change. Virtual appointments, introduced after start of the pandemic, also had substantial rates of missed appointments and cannot be viewed as the single approach that can overcome the problem of missing hospital appointments.

**Supplementary Information:**

The online version contains supplementary material available at 10.1186/s12913-023-10395-w.

## Background

The outbreak of the SARS-CoV-2 virus, coronavirus (COVID-19), was reported in the end of 2019 in Wuhan province, China, and was declared a Public Health Emergency of International Concern (PHEIC) by the World Health Organisation in January 2020 [1–3]. Since then, the virus has spread all over the world and as of August 2021, 209 million people have been infected with COVID-19, leading to 4.3 million deaths [4, 5]. Prevention measures have been taken by governments around the world to reduce the spread of the virus and the number of infections, for example, social distancing, wearing masks, washing hands, national lockdowns, and restrictions to travel have been implemented in order to reduce the spread of COVID-19 and decrease the number of new cases to prevent overburden and the collapse of our healthcare systems [6–9].

The first COVID-19 positive cases in Oman were reported on 24^th^of February 2020 for two Omani passengers coming back from Iran [10]. The number of new cases increased thereafter, reaching two peaks in July 2020 and April 2021 [11, 12]. Different measures were taken in Oman to reduce the spread of the virus. Schools, colleges, along with mosques and other public places such as beaches, playgrounds, airports and boarders were closed and national lockdowns were enforced [13–15]. The government reduced the number of employees attending their place of work in both public and private sectors to a minimum and remaining staff were required to work from home [16]. The Supplement provides further details on key dates and national lockdown periods, as well as total number of confirmed COVID-19 cases and death during the study period (Supplementary paragraph 1). In Oman, total number of confirmed COVID-19 cases was 6,370 and death was 30 by May of 2020 [10].

The spread of the virus interrupted the treatments and follow up and hospital admissions for non-COVID-19 patients, with admission declining [17–19]. The British Medical Association report stated that there was an increase in missed outpatient appointments and a decrease in elective hospital admission during April, May and June of 2020 in England [20] and a noticed reduction by 25% in A&E visits during the COVID-19 lockdown periods was reported in England [21]. Previous work by the research team found that between 2014–2019 the overall rate of missed appointment at The Royal Hospital was 22.3%, ranging from 14% to 30.3% by clinic. The study found that age, sex, service cost, waiting time, appointment day and the distance a patient lived from the hospital were important predictors for missed appointment [22]. To our knowledge, no such study has been conducted to investigate the impact of COVID-19 on the rate of missed hospital outpatient appointment in Oman.

The aims of this study were (i) to evaluate the impact of COVID-19 on the rate of missed hospital appointments in The Royal Hospital in the Oman capital city Muscat, (ii) to evaluate the impact of the restrictions measures taken by the government to reduce the rate of infection on face-to-face and virtual appointment attendance and (iii) to compare the impact of COVID-19 on the factors associated with missed appointment pre and post pandemic, providing knowledge to the health authorities.

## Methods

### Study design

A single-centred retrospective case–control design was implemented to assess outpatient attendance for multiple clinics at The Royal Hospital, Sultanate of Oman. All scheduled appointments from 1^st^ of January 2019 to 31^st^ of March 2021 were included.

### Setting

The Royal Hospital is the largest tertiary hospital, with 50 specialty and sub-specialty clinics located in the capital city Muscat of the Sultanate of Oman. The hospital provides both inpatient and outpatient medical services with 630-hospital bed capacity and 500 daily-referred patients [23]. The Royal Hospital is the main hospital in Oman providing specialised medical services that cannot be found anywhere else in the country, for example the National Cardiology Centre and the National Oncology Centre of Oman reside here. This means that the majority of treatment delivered by these subspecialty clinics is the only place where patients can access required medical services. The hospital remained open during the pandemic period with maximum staffing to provide service to patents with urgent medial needs and to patients admitted with COVID-19. Non-urgent medical services/procedures and outpatients’ appointments were cancelled and/or rescheduled to reduce patient contact and the spread of COVID-19 whilst reducing the burden on hospital staff and facilities.

### Data collection

The electronic health records (EHRs) stored in The Royal Hospital health information management system (ALSHIFA system, Version 3 +) was used to extract scheduled outpatient clinics appointment data.

### Study sample

There were 343,149 scheduled appointments included in the final study sample divided into 320,049 face-to-face and 23,100 virtual clinic appointments. Supplementary Fig. 1 shows the exclusion process for the face-to-face appointments. A total of 2,121 observations was excluded from the initial dataset abstracted from the hospital health information management system (ALSHIFA) due to duplicated records, missing data for age, governorate, and marital status; outlier age for the Paediatric clinic (age > 18 years) and the Obstetrics and Gynaecology clinic (age < 13 years); appointments recorded on a Saturday (not a working day) and unidentified gender.

The face-to-face sample was divided into three datasets, pre COVID-19 (*N* = 178,849), between (*N* = 11,516) and post start of COVID-19 (*N* = 129,684). All scheduled appointment from the 1^st^ of January 2019 to the first reported case of COVID-19 (24^th^ February 2020) was grouped as pre COVID-19. The observations from the date when the first cases were reported until the date when the government started to implement restriction and measures to contain and reduce the spread of the virus (1^st^of April 2020) were grouped as between COVID-19 and the observations after the implementation of the first restriction measure were grouped as post COVID-19. This process is in line with similar published studies [24, 25].

The virtual clinic appointments were introduced post COVID-19 from 16^th^ of April 2020. The face-to-face scheduled appointments for pre and post COVID-19 periods were used in the analysis with a total of 308,533 observations. These observations were further divided into one dataset including appointments scheduled within the Paediatric clinic only (*N* = 79,444), another dataset including Obstetrics and Gynaecology appointment only (*N* = 51,850) and an overall dataset including scheduled appointments from all remaining outpatient clinics (*N*= 177,239). Our previous work observed large variability in the rate of missed appointments by clinic [22]. Therefore, the top five clinics with highest number of scheduled appointments during the study period and higher rate of missed appointments compared to other clinics were identified and five subsets of data created, including: Diabetes and Endocrine clinic (*N* = 42,238), Surgery clinic (*N* = 22,781), Oncology clinic (*N* = 20,850), Urology clinic (*N* = 11,620), and Gastroenterology clinic (*N*= 7,946). The detailed sampling procedure is documented here [22].

### Measures

A missed appointment was defined as a patient who had a scheduled appointment but did not attend the appointment without contacting the hospital to cancel or rebook the appointment. This is recorded in the system as failed to attend, in line with other research studies [26]. Our previous study showed that older patients (65 +), living further away from the hospital, that waited for more than 60 days, and patients paying visit and registration fees were more likely to miss their hospital appointments [22]. The same predictors were adopted in the analysis for this study.

### Statistical analysis

Patient demographic characteristics were summarised for the overall population, by appointment status and stratified by the time period pre and post COVID-19 (face-to-face and virtual clinic appointments). Logistic regression models were used to estimate odd ratios (OR) and 95% confidence intervals (CI) for a binary outcome of hospital appointment status (attended and missed). According to many studies, logistic regression is the most widely used technique for modelling a binary outcome [27]. Interaction term analysis was performed for the binary outcome (pre and post COVDI-19 and post COVID-19 face-to-face and post COVID-19 virtual clinic appointments). Models were fitted separately for face-to-face appointments for the overall dataset and stratified by clinic, for the Paediatric clinic, for the Obstetrics and Gynaecology clinic, and for each of the top five clinics with highest number of scheduled appointments. For the virtual clinic appointment one overall model was fitted. The R statistical program (version 3.6.2) was used for the statistical analyses [28]. All methods were carried out in accordance with relevant guidelines and regulations. The study was approved by the Study and Research Centre, Ministry of Health, Sultanate of Oman on 2 May 2019 (proposal ID: MoH/CSR/20/24172).

## Results

There were 343,149 scheduled outpatient appointments included in the analysis, divided into 320,049 face-to-face appointments for the time period between 1^st^ of January 2019 and 31^st^ of March2021 and 23,100 virtual clinic appointment for the time period between 16^th^ of April 2020 and 31^st^ of March 2021. A total of 63,257 face-to-face appointments (19.8%) was missed. For face-to-face appointments included in the study, 116,859 (36.5%) appointments were for male patients and 72,780 (22.7%) were for patients aged between 31 and 40 years old (Table 1). Additional baseline characteristics of the study population can be found in Supplementary Tables 1 and 2.

**Table 1:** Characteristics of the study population for each unique patient, for all scheduled appointments, stratified by attended or missed face-to-face appointments

	**Characteristics of Patients (N)**	**Characteristics of Appointments (N)**	**Attended** **N (%)**	**Missed** **N (%)**
	80,835	320,049	256,792 (80.2)	63,257 (19.8)
**Sex**			*p value < 0.001	
Female	48473 (60.0)	203190 (63.5)	165475 (81.4)	37715 (18.6)
Male	32362 (40.0)	116859 (36.5)	91317 (78.1)	25542 (29.1)
**Age category**			*p value < 0.001	
≤ 5 years old	12667 (15.7)	36823 (11.5)	29260 (79.5)	7563 (20.5)
6–10 years old	5247 (6.5)	21850 (6.8)	16738 (76.6)	5112 (23.4)
11–13 years old	2426 (3.0)	11901 (3.7)	8768 (73.7)	3133 (26.3)
14–18 years old	2336 (2.9)	12243 (3.8)	8942 (73.0)	3301 (27.0)
19–30 years old	11192 (13.8)	42312 (13.2)	35437 (83.8)	6875 (16.2)
31–40 years old	17118 (21.2)	72780 (22.7)	60481 (83.1)	12299 (16.9)
41–50 years old	10953 (13.5)	46758 (14.6)	37129 (79.4)	9629 (20.6)
51–60 years old	7546 (9.3)	31538 (9.9)	24865 (78.8)	6673 (21.2)
61–70 years old	6515 (8.1)	26207 (8.2)	20936 (79.9)	5271 (20.1)
71–80 years old	3609 (4.5)	13497 (4.2)	10871 (80.5)	2626 (19.5)
> 80 years old	1226 (1.5)	4140 (1.3)	3365 (81.3)	775 (18.7)
**Appointment day**			*p value < 0.001	
Sunday	16107 (19.9)	62174 (19.4)	50433 (81.1)	11741 (18.9)
Monday	16544 (20.5)	66185 (20.7)	53074 (80.2)	13111 (19.8)
Tuesday	17455 (21.6)	71785 (22.4)	57259 (79.8)	14526 (20.2)
Wednesday	16456 (20.4)	63954 (20.0)	51234 (80.1)	12720 (19.9)
Thursday	14273 (17.7)	55951 (17.5)	44792 (80.1)	11159 (19.9)
**Governorate**			*p value < 0.001	
Muscat	41922 (51.9)	169790 (53.1)	139254 (82.0)	30536 (18.0)
South Batina	1298 (1.6)	4482 (1.4)	3392 (75.7)	1090 (24.3)
AL Dhakiliya	524 (0.6)	1792 (0.6)	1305 (72.8)	487 (27.2)
North Batina	1140 (1.4)	4190 (1.3)	3226 (77.0)	964 (23.0)
North Sharqiya	6951 (8.6)	28801 (9.0)	22630 (78.6)	6171 (21.4)
South Sharqiya	7069 (8.7)	29342 (9.2)	22875 (78.0)	6467 (22.0)
AL Dhahira	8461 (10.5)	32115 (10.0)	25918 (80.7)	6197 (19.3)
AL Buriami	5144 (6.4)	17932 (5.6)	13665 (76.2)	4267 (23.8)
AL Wusta	4612 (5.7)	17144 (5.4)	13409 (78.2)	3735 (21.8)
Musandam	3311 (4.1)	13191 (4.1)	10143 (76.9)	3048 (23.1)
Dhofar	390 (0.5)	1222 (0.4)	942 (77.1)	280 (22.9)
GCC Countries^a^	13 (0.0)	48 (0.0)	33 (68.8)	15 (31.2)
**Service cost**			*p value < 0.001	
Pay visit and registration fees	66172 (81.9)	269269 (84.1)	216503 (80.4)	52766 (19.6)
Pay all medical service fees	3310 (4.1)	12620 (3.9)	10328 (81.8)	2292 (18.2)
≤ 2 years old^b^	7754 (9.6)	19775 (6.2)	15871 (80.3)	3904 (19.7)
Under Social Affair coverage^b^	3599 (4.5)	18385 (5.7)	14,090 (76.6)	4295 (23.4)
**Appointment waiting days**			*p value < 0.001	
≤ 30 days	26240 (32.5)	118479 (37.0)	100454 (84.8)	18025 (15.2)
31—60 days	11732 (14.5)	44067 (13.8)	36351 (82.5)	7716 (17.5)
61—90 days	8136 (10.1)	32944 (10.3)	25427 (77.2)	7517 (22.8)
91—120 days	9004 (11.1)	37992 (11.9)	29202 (76.9)	8790 (23.1)
> 120 days	25723 (31.8)	86567 (27.0)	65358 (75.5)	21209 (24.5)

^*^p value calculated using the Tableone R package which summarises categorical and continuous variables. The chi-squared test was used to calculate the *p* value for categorical variable and one-way analysis of variance (ANOVA) for continuous variables

^a^The Cooperation Council for the Arab States of the Gulf

^b^Exempted from all medical service fees. Between 1^st^ Jan 2019 – 31^st^ March 2021, for regression analysis 11,516 observations were removed from the between covid-19 period. For additional characteristics see supplementary table 1

**Fig. 1:**
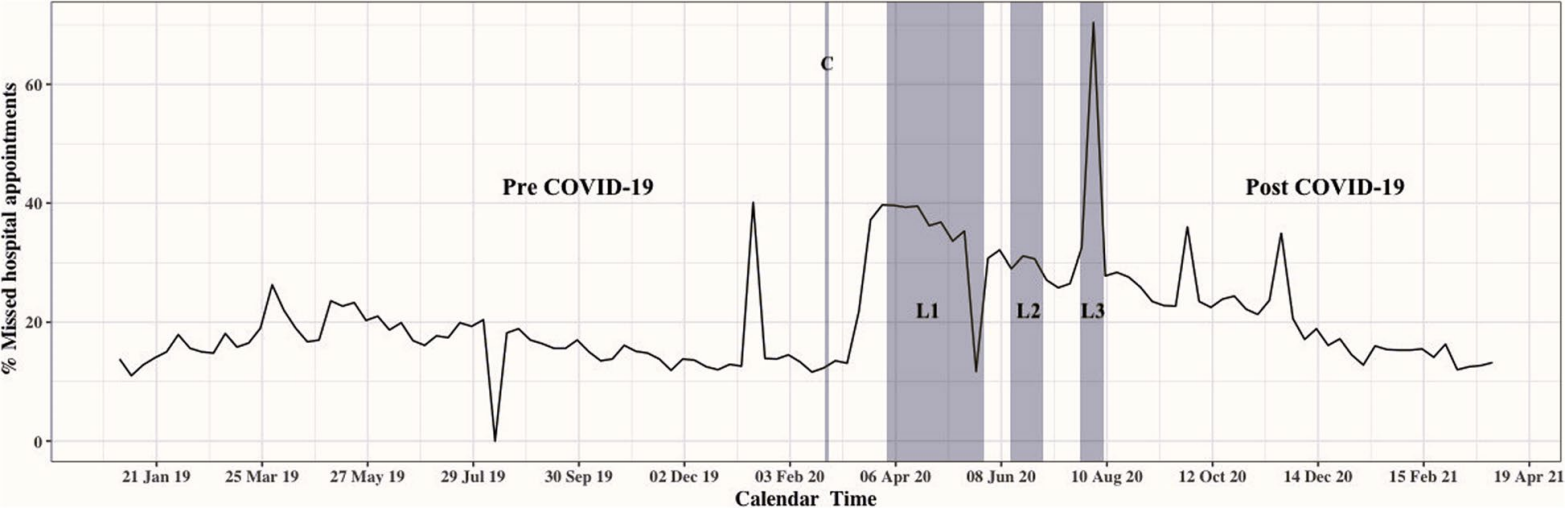
Weekly rate of missed face-to-face hospital appointments over calendar time at The Royal Hospital outpatient clinics. C: The first confirmed case of COVID-19 in Oman (24–2-2020). L1: Lockdown period 1 (from 1/4/2020 to 29/5/2020). L2: Lockdown period 2 (from 13/6/2020 to 3/7/2020). L3: Lockdown period 3 (from 25/7/2020 to 8/8/2020). Lockdown periods included the restriction of movement between governorates, except for emergency situations and patients with a hospital appointment. Lockdown period 3 included a public holiday with a reduction in scheduled appointments for three days (2–3-4/8/2020). The drop shown in August 2019 (Pre COVID-19) correlated to weeks public holiday (with no scheduled appointments). The increase shown in January 2020 (Pre COVID-19) correlated to the three days of mourning following the death of Sultan Qaboos bin Said (two days of scheduled appointments only). For data underlying the plot see Supplementary Table 3

The face-to-face pre and post COVID-19 analysis showed that the number of scheduled appointments declined by 27.5% from pre to post COVID-19 (Table 2). However, the rate of missed hospital appointments rose from 16.9% pre to 23.8% post COVID-19 (OR of 1.72 [95% CI 1.68–1.76] comparing post to pre COVID-19). When looking at the weekly rates of missed appointments overall, an increase in the rate of missed hospital appointments was observed when the first lockdown was implemented from 10 April 2020 to 21 June 2020, raising from 19.2% to 40.5%. Similarly, an increase in the rate of missed appointments was seen in the second and third lockdown periods (Fig. 1). Supplementary Table 3 provides details about the distribution and frequency of appointments during lockdown periods.

**Table 2:** Case mix and rate of missed face-to-face appointments pre and post COVID-19 for all outpatient clinics at The Royal Hospital

	**Pre COVID-19 N (%)** 178,849 (58.0)	**Post COVID-19 N (%)** 129,684 (42.0)
**Appointment status**		
Attend	148693 (83.1)	98788 (76.2)
Missed	30156 (16.9)	30896 (23.8)
**Sex**		
Female	112382 (62.8)	83474 (64.4)
Male	66467 (37.2)	46210 (35.6)
**Age category**		
≤ 5 years old	21486 (12.0)	13939 (10.7)
6–10 years old	11808 (6.6)	9210 (7.1)
11–13 years old	6251 (3.5)	5158 (4.0)
14–18 years old	6593 (3.7)	5199 (4.0)
19–30 years old	24183 (13.5)	16588 (12.8)
31–40 years old	40200 (22.5)	30017 (23.1)
41–50 years old	26154 (14.6)	18979 (14.6)
51–60 years old	17895 (10.0)	12501 (9.6)
61–70 years old	14543 (8.1)	10796 (8.3)
71–80 years old	7513 (4.2)	5535 (4.3)
> 80 years old	2223 (1.2)	1596 (1.4)
**Appointment day**		
Sunday	35093 (19.6)	25090 (19.3)
Monday	36421 (20.4)	27700 (21.4)
Tuesday	39019 (21.8)	29949 (23.1)
Wednesday	35382 (19.8)	26063 (20.1)
Thursday	32934 (18.4)	20882 (16.1)
**Governorate**		
Muscat	94574 (52.9)	69196 (53.4)
South Batina	2683 (1.5)	1651 (1.3)
AL Dhakiliya	1123 (0.6)	607 (0.5)
North Batina	2205 (1.2)	1833 (1.4)
North Sharqiya	15995 (8.9)	11748 (9.1)
South Sharqiya	16301 (9.1)	11957 (9.2)
AL Dhahira	18130 (10.1)	12789 (9.9)
AL Buriami	10178 (5.7)	7160 (5.5)
AL Wusta	9653 (5.4)	6879 (5.3)
Musandam	7246 (4.1)	5405 (4.2)
Dhofar	729 (0.4)	443 (0.3)
GCC Countries^a^	32 (0.0)	16 (0.0)
**Service cost**		
Pay visit and registration fees	149545 (83.6)	110194 (85.0)
Pay all medical service fees	7251 (4.1)	4856 (3.7)
≤ 2 years old	11623 (6.5)	7391 (5.7)
Under Social Affair coverage^b^	10430 (5.8)	7243 (5.6)
**Appointment waiting days**		
≤ 30 days	59701 (33.4)	54531 (42.0)
31- 60 days	26073 (14.6)	16381 (12.6)
61–90 days	20514 (11.5)	11365 (8.8)
91- 120 days	23497 (13.1)	13243 (10.2)
> 120 days	49064 (27.4)	34164 (26.3)
**Marital status**		
Married	102824 (57.5)	74481 (57.4)
Child	39545 (22.1)	28307 (21.8)
Divorced	829 (0.5)	533 (0.4)
Single	34528 (19.3)	25563 (19.7)
Widow	1123 (0.6)	800 (0.6)

^a^The Cooperation Council for the Arab States of the Gulf

^b^Exempted from all medical service fees, Pre COVID-19 from (1/1/2019 to 23/2/2020) Post COVID-19 from (1/4/2020 to 31/3/2021)

The rates of missed appointments increased post COVID-19 in most clinics. In the Paediatric clinic, it increased from 19.3% pre to 28.2% post COVID-19 and in the Surgery clinic from 12.5% to 25.5%. The only exception was the Obstetrics & Gynaecology clinic in which rates remained similar (8.4% to 8.5%). More details are given in Supplementary Fig. 2.

Table 3 shows the ORs for predictors and the change in their effect pre and post COVID-19 (interaction analyses). Most predictors of missed appointments showed no substantive changes in effect after COVID-19 compared to before (i.e., interaction term not statistically significant), except for waiting time and appointment day. The effect of waiting time increased pre to post COVID-19 for all waiting time groups from 1.38 (95% CI 1.31 to 1.46) pre COVID-19 to 2.47 (95% CI 2.33 to 2.62) post COVID-19 for waiting time between 91–120 days. This effect was greater for patients with a waiting time > 120 days, from 1.46 (95% CI 1.39 to 1.53) pre COVID-19 to 2.68 (95% CI 2.56 to 2.80) post COVID-19. In contrast, the day of the scheduled appointment had opposite effects pre and post COVID-19. For example, appointments scheduled on Mondays were less likely to be missed pre COVID-19 [OR 0.94 (95% CI 0.89 to 0.99)] but missed post COVID-19 [OR 1.24 (95% CI 1.17 to 1.31)] for all clinics except Obstetrics & Gynaecology and Paediatric clinics; a similar pattern was observed for appointments scheduled on Thursdays. The effect of age and sex on missed appointment was proportionally similar pre and post COVID-19. For patients aged 41–50 the adjusted OR was 1.04 (95% CI 0.99 to 1.09) pre COVID-19 and 0.95 (95% CI 0.90–1.00) post COVID-19; interaction term was not statistically significant (adjusted OR 0.96 (95% CI 0.89–1.02)).

**Table 3:** Predictors of missed face-to-face hospital appointments at The Royal Hospital outpatient clinics (excluding Obstetrics & Gynaecology and Paediatric clinics) pre and post COVID-19

	**Pre COVID-19** (*N* = 103,393)	**Post COVID19** (*N* = 73,846)	^**c**^** Interaction** (*N* = 177,239)
	**OR (95% CI)**	**OR (95% CI)**	**OR (95% CI)**
**Sex**			
Female	**Reference**		
Male	1.07 (1.03–1.11)^b^	0.99 (0.96–1.03)	0.99 (0.95–1.04)
**Age category**			
19–30 years old	0.96 (0.91–1.01)	0.96 (0.90–1.02)	0.99 (0.92–1.07)
31–40 years old	**Reference**		
41–50 years old	1.04 (0.99–1.09)	0.95 (0.90–1.00)^b^	0.96 (0.89–1.02)
51–60 years old	0.98 (0.93–1.03)	0.96 (0.91–1.02)	1.05 (0.98–1.13)
61–70 years old	0.94 (0.89–1.00)^b^	0.94 (0.89–1.00)^b^	1.04 (0.96–1.12)
71–80 years old	0.93 (0.86–1.00)^b^	0.95 (0.88–1.02)	1.04 (0.94–1.15)
> 80 years old	0.84 (0.74–0.95)	0.92 (0.81–1.04)	1.10 (0.93–1.30)
**Appointment day**			
Sunday	**Reference**		
Monday	0.94 (0.89–0.99)^b^	1.24 (1.17–1.31)^b^	1.40 (1.30–1.51)^b^
Tuesday	1.03 (0.98–1.08)	1.12 (1.06–1.19)^b^	1.11 (1.03–1.19)^b^
Wednesday	0.94 (0.89–0.99)^b^	1.16 (1.10–1.22)^b^	1.18 (1.10–1.27)^b^
Thursday	0.97 (0.92–1.03)	1.19 (1.12–1.26)^b^	1.26 (1.17–1.36)^b^
**Service cost**			
Pay visit and registration fees	**Reference**		
Pay all medical service fees	0.81 (0.66–1.00)^b^	0.86 (0.69–1.06)	1.09 (0.97–1.23)
Under Social Affair coverage^a^	1.00 (0.94–1.07)	1.08 (1.01–1.16)^b^	1.09 (0.99–1.20)
**Appointment waiting days**			
≤ 30 days	**Reference**		
31–60 days	1.35 (1.28–1.42)^b^	2.02 (1.91–2.14)^b^	1.47 (1.36–1.59)^b^
61–90 days	1.46 (1.38–1.54)^b^	2.39 (2.25–2.54)^b^	1.71 (1.58–1.84)^b^
91–120 days	1.38 (1.31–1.46)^b^	2.47 (2.33–2.62)^b^	1.87 (1.74–2.02)^b^
> 120 days	1.46 (1.39–1.53)^b^	2.68 (2.56–2.80)^b^	1.84 (1.74–1.96)^b^
**Marital status**			
Married	**Reference**		
Single	1.05 (1.00–1.09)^b^	0.97 (0.93–1.01)	0.96 (0.91–1.01)
Divorced	0.93 (0.76–1.13)	0.96 (0.78–1.18)	1.16 (0.87–1.53)
Widow	1.19 (1.02–1.39)^b^	0.96 (0.81–1.14)	0.86 (0.69–1.08)
**Nationality**			
Omani	**Reference**		
Non-Omani	1.24 (1.01–1.54)^b^	1.18 (0.95–1.48)	1.06 (0.94–1.20)

^a^Exempted from all medical service fees

^b^ Statistically significant

^c^Interaction: model adjusted with an interaction term for Pre and Post COVID-19

Distance of patient residence to the hospital also showed no major changes in relative effect pre and post COVID-19 (Supplementary Table 4). For Dhofar governorate (800–1120 km from Royal hospital), the adjusted OR was 1.15 (95% CI 0.90 to 1.48) pre COVID-19 and 1.01 (95% CI 0.75 to 1.35) post COVID-19; interaction term was not statistically significant (OR 0.97 (95% CI 0.66 to 1.41)). Supplementary Tables 5–11 shows the results of logistic regression analysis pre and post COVID-19 in the top five clinics, Obstetrics & Gynaecology, and Paediatric clinics.

There were 23,100 virtual clinics appointments from the start of the service in 16^th^ of April 2020 to the 31^st^ of March 2021 scheduled for 11,437 patients. A total of 7,447 appointments (32.2%) was missed. For all virtual appointments, 15,341 (66.4%) appointments were for female patients and 5,326 (23.1%) were for patients aged between 31 and 40 years old (Table 4). Additional baseline can be found in Supplementary Tables 12 and 13.

**Table 4:** Characteristics of the study population for each unique patient, for all scheduled appointments, stratified by attended or missed for virtual clinics appointments

	**Characteristics of Patients (N)** 11,437	**Characteristics of Appointments (N)** 23,100	**Attended** **N (%)** 15,653 (67.8)	**Missed** **N (%)** 7,447 (32.2)
**Sex**			*p value < 0.001	
Female	7129 (62.3)	15341 (66.4)	10087 (65.8)	5254 (34.2)
Male	4308 (37.7)	7759 (33.6)	5566 (71.7)	2193 (28.3)
**Age category**			*p value < 0.001	
≤ 5 Years old	792 (6.9)	1316 (5.7)	1036 (78.7)	280 (21.3)
6–10 years old	928 (8.1)	1529 (6.6)	1199 (78.4)	330 (21.6)
11–13 years old	504 (4.4)	1065 (4.6)	756 (71.0)	309 (29.0)
14–18 years old	664 (5.8)	1507 (6.5)	1016 (67.4)	491 (32.6)
19–30 years old	1174 (10.3)	2680 (11.6)	1683 (62.8)	997 (37.2)
31–40 years old	2220 (19.4)	5326 (23.1)	3363 (63.1)	1963 (36.9)
41–50 years old	2015 (17.6)	4094 (17.7)	2689 (65.3)	1405 (34.7)
51–60 years old	1362 (11.9)	2443 (10.6)	1663 (68.1)	780 (31.9)
61–70 years old	1059 (9.3)	1981 (8.6)	1395 (70.5)	586 (29.5)
71–80 years old	526 (4.6)	851 (3.7)	620 (72.9)	231 (27.1)
> 80 Years old	193 (1.7)	308 (1.3)	233 (75.6)	75(24.4)
**Appointment day**			*p value < 0.001	
Sunday	2052 (17.9)	4201 (18.2)	2843 (67.7)	1358 (32.3)
Monday	3321 (29.0)	6180 (26.8)	4326 (70.0)	1854 (30.0)
Tuesday	2286 (20.0)	4850 (21.0)	3366 (69.9)	1484 (30.1)
Wednesday	2396 (20.9)	5032 (21.8)	3163 (62.9)	1869 (37.1)
Thursday	1382 (12.1)	2837 (12.3)	1955 (68.9)	882 (31.1)
**Governorate**			*p value < 0.001	
Muscat	5754 (50.3)	12,609 (54.6)	8206 (65.1)	4403 (34.9)
South Batina	1277 (11.2)	2212 (9.6)	1627 (73.6)	585 (26.4)
AL Dhakiliya	1091 (9.5)	2192 (9.5)	1529 (69.9)	663 (30.1)
North Batina	957 (8.4)	1805 (7.8)	1248 (69.1)	557 (30.9)
North Sharqiya	664 (5.8)	1202 (5.2)	840 (69.9)	362 (30.1)
South Sharqiya	754 (6.6)	1359 (5.9)	979 (72.1)	380 (27.9)
AL Dhahira	459 (4.0)	852 (3.7)	608 (71.4)	244 (28.6)
AL Buriami	175 (1.5)	319 (1.4)	221 (69.3)	98 (30.7)
AL Wusta	47 (0.4)	75 (0.3)	59 (78.7)	16 (21.3)
Musandam	62 (0.5)	136 (0.6)	92 (67.7)	44 (32.3)
Dhofar	194 (1.7)	334 (1.4)	242 (72.5)	92 (27.5)
GCC Countries^a^	3 (0.0)	5 (0.0)	2 (40.0)	3 (60.0)
**Service cost**			*p value < 0.001	
Pay visit and registration fees	10,877 (95.1)	22,131 (95.8)	15,057 (68.0)	7074 (32.0)
Pay all medical service fees	254 (2.2)	387 (1.7)	204 (52.7)	183 (47.3)
≤ 2 years old^b^	303 (2.6)	573 (2.5)	385 (67.2)	188 (32.8)
Under Social Affair coverage^b^	3 (0.0)	9 (0.0)	7 (77.8)	2 (22.2)
**Appointment waiting days**			*p value < 0.001	
≤ 30 days	8349 (73.0)	17,669 (76.5)	12,160 (68.8)	5509 (31.2)
31- 60 days	1445 (12.6)	2468 (10.7)	1688 (68.3)	784 (31.7)
61–90 days	782 (6.8)	1389 (6.0)	905 (65.2)	482 (34.8)
91- 120 days	633 (5.5)	1171(5.1)	699 (59.8)	469 (40.2)
> 120 days	228 (2.0)	403 (1.7)	196 (48.5)	208 (51.5)

^*^p value calculated using the Tableone R package which summarises categorical and continuous variables. The chi-squared test was used to calculate the *p* value for categorical variable and one-way analysis of variance (ANOVA) for continuous variables

^a^The Cooperation Council for the Arab States of the Gulf

^b^Exempted from all medical service fees. For additional characteristics see supplementary table 12

**Fig. 2:**
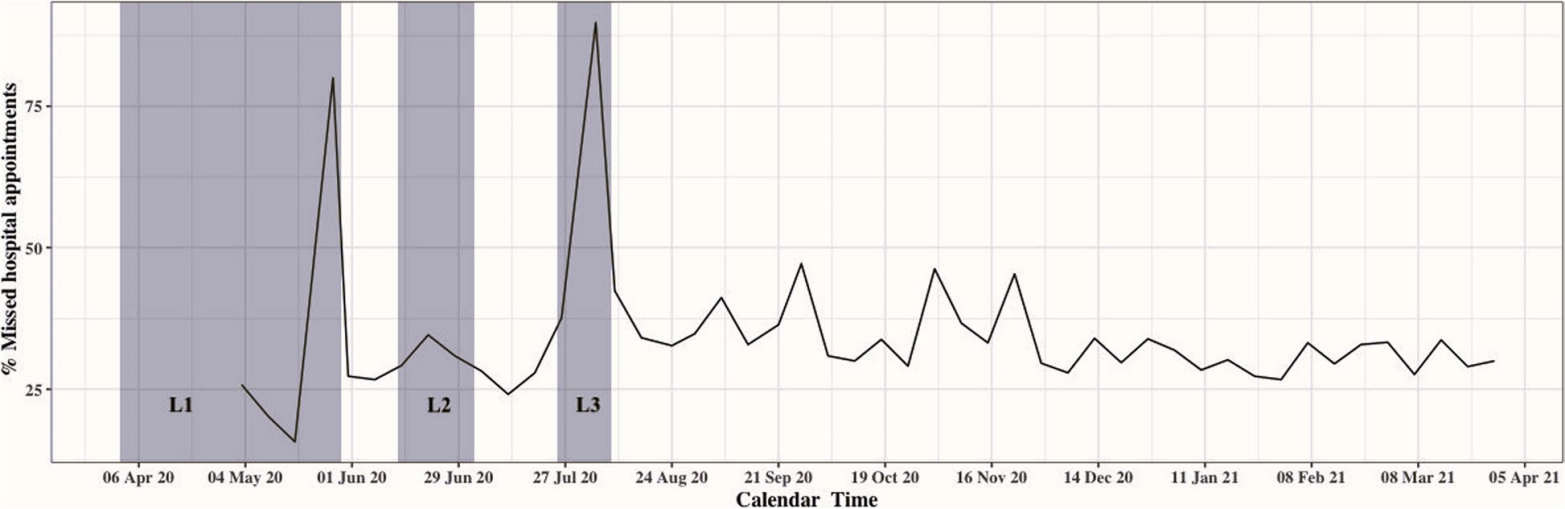
Weekly rate of missed virtual clinic appointments over calendar time at The Royal Hospital. The first recorded virtual clinic appointment in (16–04-2020), with just one scheduled appointment. L1: Lockdown period 1 (from 1/4/2020 to 29/5/2020). L2: Lockdown period 2 (from 13/6/2020 to 3/7/2020). L3: Lockdown period 3 (from 25/7/2020 to 8/8/2020). Lockdown periods included the restriction of movement between governorates, except for emergency situations and patients with a hospital appointment. Lockdown period 3 included a public holiday with a reduction in scheduled appointments for three days (2–3-4/8/2020). For data underlying the plot see Supplementary Table 3

When looking at the weekly rates of missed virtual clinic appointments overall, an increase in the rate of missed appointments was observed in all lockdown periods (Fig. 2). See supplementary Table 3 for more details about the distribution and frequency of virtual clinic appointments during lockdown periods.

Table 5 shows the ORs for predictors and change in their effect post COVID-19 face-to-face and virtual appointments (interaction analyses). Distance of patient residence to the hospital also showed no major changes in relative effect post COVID-19 face-to-face and post COVID-19 virtual appointments (Supplementary Table 14).

**Table 5:** Predictors of missed hospital appointments post COVID-19 at The Royal Hospital outpatient clinics (excluding Obstetrics & Gynaecology and Paediatric clinics) for face-to-face and virtual appointments

	**Post COVID-19 Face-to-Face** (*N* = 73,846)	**Post COVID-19 Virtual** (*N* = 16,432)	^**a**^**Interaction** (*N* = 90,278)
	**OR (95% CI)**	**OR (95% CI)**	**OR (95% CI)**
**Sex**			
Female	**Reference**		
Male	0.99 (0.96–1.03)	0.84 (0.78–0.91)^b^	0.95 (0.93–0.99)^b^
**Age Category**			
19–30 years old	0.96 (0.90–1.02)	1.08 (0.96–1.20)	0.95 (0.90–1.00)
31–40 years old	**Reference**		
41–50 years old	0.95 (0.90–1.00)	0.84 (0.77–0.92)^b^	0.88 (0.84–0.92)^b^
51–60 years old	0.96 (0.91–1.02)	0.73 (0.65–0.81)^b^	0.83 (0.79–0.87)^b^
61–70 years old	0.94 (0.89–1.00)	0.67 (0.59–0.75)^b^	0.76 (0.72–0.80)^b^
71–80 years old	0.95 (0.88–1.02)	0.59 (0.49–0.69)^b^	0.73 (0.68–0.78)^b^
> 80 years old	0.92 (0.81–1.04)	0.54 (0.41–0.71)^b^	0.69 (0.61–0.77)^b^
**Appointment day**			
Sunday	**Reference**		
Monday	1.24 (1.17–1.31)^b^	0.78 (0.70–0.87)^b^	1.21 (1.15–1.26)^b^
Tuesday	1.12 (1.06–1.19)^b^	0.71 (0.64–0.80)^b^	1.06 (1.01–1.11)^b^
Wednesday	1.16 (1.10–1.22)^b^	1.03 (0.93–1.15)	1.13 (1.08–1.19)^b^
Thursday	1.19 (1.12–1.26)^b^	0.65 (0.57–0.73)^b^	1.06 (1.00–1.11)^b^
**Appointment waiting days**			
≤ 30 days	**Reference**		
31–60 days	2.02 (1.91–2.14)^b^	1.22 (1.09–1.36)^b^	1.68 (1.60–1.77)^b^
61–90 days	2.39 (2.25–2.54)^b^	1.26 (1.10–1.45)^b^	2.08 (1.97–2.19)^b^
91–120 days	2.47 (2.33–2.62)^b^	1.76 (1.52–2.03)^b^	2.16 (2.05–2.27)^b^
> 120 days	2.68 (2.56–2.80)^b^	3.46 (2.69–4.45)^b^	2.46 (2.36–2.56)^b^
**Marital status**			
Married	**Reference**		
Single	0.97 (0.93–1.01)	0.86 (0.78–0.94)	0.99 (0.95–1.02)
Divorced	0.96 (0.78–1.18)	0.89 (0.58–1.37)	1.03 (0.86–1.25)
Widow	0.96 (0.81–1.14)	0.72 (0.48–1.08)	0.96 (0.82–1.12)
**Nationality**			
Omani	**Reference**		
Non-Omani	1.18 (0.95–1.48)^b^	1.47 (1.25–1.72)^b^	1.01 (0.93–1.11)

^a^Interaction: model adjusted with an interaction term for Post COVID-19 Face–to-Face appointments and Post COVID-19 virtual appointments

^**b**^Statistically significant

## Discussion

This study found a substantive decrease in the number of scheduled appointments after start of the COVID-19 pandemic in Oman while the rate of missed appointments increased substantially. This increase in missed appointments rates occurred in almost all clinics except for the Obstetrics & Gynaecology clinic that had comparable rates pre and post COVID-19. Rates of missed virtual appointments, introduced after the start of the pandemic, were also high.

The overall increase in rate of missed appointments observed in our study could be linked to the restrictions enforced in Oman to control the spread of the virus. The increases observed were associated with the time when restriction of movement was implemented by the government and the highest weekly increase was noticed in the same week the first national lockdown was enforced across Oman. Different studies had reported an increase in the rate of missed hospital appointment during COVID-19 lockdowns [29–31]. The study also showed a decline in the overall number of scheduled appointments within the outpatient clinics during the COVID-19 pandemic, which is consistent with reports from other published studies [32, 33]. This can be explained by the measures taken by the hospital to protect patients and medical staffs by cancelling non urgent appointments and reducing the number of newly booked appointments, only offering them to patients with critical needs [34].

Furthermore, the decline in the scheduled appointments and increase in the rate of missed appointments observed during the COVID-19 pandemic could be related to the patients fear to be infected with the virus causing them to skip their hospital appointments without notifying the hospital. All announcements regarding the rate of COVID-19 infection in Oman, including new case rates, COVID-19 related hospital admissions and COVID-19 related deaths were published and broadcasted in a weekly news conference by the authorities (COVID-19 Supreme Committee) [35]. Patients decisions to attend their hospital appointments may have been influenced by these announcements, including an increased fear of acquiring COVID-19 from attending the hospital, which could also explain the increase in the rate of missed appointments noticed during the post COVID-19. Similar reports from different studies documented the same reasons for the increase in missed hospital appointments [35–37]. Our previous work looking at the reasons for missed hospital appointments at The Royal Hospital between March and April of 2021 and showed that some patients did not attend because they were afraid of getting infected with the virus [38]. As this study was conducted almost a year after the first cases of COVID-19 were reported in Oman, it indicates this fear may still be prevalent in the post COVID-19 period.

Variability in the missed appointment rate was found in the top outpatient clinics. The nature of the service and severity of illness may explain the variability in rate of missed appointment by clinic. Similar published studies have reported variability in the rate of missed appointment within different clinics due to the difference in type of disease and the required medical services, especially during the COVID-19 pandemic [39–42]. The rate of missed appointment in the Oncology clinic and Obstetrics and Gynaecology were low compared to other clinics, where as some clinics such as Paediatric and Surgery, there was a large increase in missed appointment rates.

The Royal Hospital is the only health facility providing oncology treatment in Oman and no such service is available elsewhere, suggesting the rate of missed appointments for this clinic is less because patients have no other location to receive vital treatment. The Obstetrics and Gynaecology clinic in the Royal Hospital is advanced compared to other Obstetrics and Gynaecology clinics in other hospitals. It is staffed with more consultants and specialties trained medical staff. As a result, patients may prefer to have their antenatal, postnatal and other gynaecological care within the Royal Hospital. Published papers found that patients would miss less appointments if they feel that they receive a good quality services [43, 44]. Unlike oncology services, patients can seek medical service for Urology and Gastroenterology clinics in private hospitals. As a result, more patients may miss these appointments because they can find the same service in private sector. In addition, given the age of the patients and likely in the early onset of disease, paediatric patients may have missed their appointments during the pandemic as they may be more able to wait for a follow-up appointment once restrictions lifted, whereas older patients in later stages of disease and increased severity may not be able to wait for treatment.

When comparing the characteristics of patient’s pre and post COVID-19 there were no significant changes. It was expected that more vulnerable patients (e.g., the elderly and or patients of poor health) would be more likely to miss their appointments post COVID-19 compared to pre COVID-19 because they were more at high risk of complications if they caught COVID-19. However, case mix analysis showed no shift in characteristics of patients’ pre and post COVID-19. This indicates factors related to patients’ characteristics may have more influence in their attendance behaviour than external factors such as COVID-19 pandemic [45]. This finding is different from findings in other published studies, which reported that older patients were more likely to miss their appointments post compared to pre COVID-19 [46, 47]. Indeed, these studies suggested that older patients were more likely to have multiple health problems and therefore, more likely to fear being infected with the virus which might lead to worsen their medical conditions. Our finding may be explained by different cultural practice in Oman where elderly patients typically live with their families, meaning they have better support for getting to and attending their hospital appointments.

Our study also showed that longer waiting time was a strong predictor for missed hospital appointment. Patients with longer waiting time tended to miss more compared to patients with short waiting time. This finding is supported by other studies that showed similar results [48, 49]. This could be explained by the fact that patients with longer waiting time might seek health services in private hospitals or other government hospitals since a patient can schedule additional appointments within other hospitals without notifying The Royal Hospital. We found that the effect of appointment day was different pre and post COVID-19. Appointments scheduled in the beginning of the week (Monday) and end of the week (Thursday) were less likely to be missed pre COVID-19 compared to appointments scheduled on other days of the week. In contrast other studies found appointments at the beginning and end of the working week were more likely to be missed [50–52]. Patients have to travel quite some distance for appointments in the capital which may be challenging, particularly if they lack transportation. These patients may share transportation with family or friends who are traveling to the capital for a working week and therefore less likely to miss an appointment at the beginning the week. On the other hand, there was a shift in the effect of weekday on missed hospital appointment rate post COVID-19. Appointments scheduled on Monday and Thursday were more likely to be missed compared to other days of the week. The change post COVID-19 is expected given the restrictions of travel to and from the capital and the enforcement for employees to work from home.

In March 2020 The Royal Hospital introduced the idea of “virtual clinics” [53]. Patients with follow up outpatient appointments for some clinics were given the option to attend their appointment via a phone call instead of face-to-face. Several published studies have shown that there was an increase in the usage of telehealth/telemedicine technology during the COVID-19 pandemic compared to the pre pandemic period. The usage of such technology helped the health care system to continue providing medical service during lockdowns and COVID-19 restriction implemented by governments to reduce the spread of the virus [54–59]. It could be expected that the possibility of virtual clinics and choice for patients would have reduced the rate of missed appointments for face-to-face clinics. However, our findings of substantially raised rates of missed virtual appointments in the Royal Hospital suggest that virtual appointments may not be the single solution to reducing missed appointments. The higher rate of missed hospital appointment for the virtual clinics appointments (32.2%) compared to face-to-face appointment pre and post COVID-19 (16.9 and 23.8 respectively) can be related to many factors. Virtual clinics were used for the first time during COVID-19 as an alternate method of delivering normal outpatient care. Also, there was no preliminary planning for the introduction of such a service, and patients might not have received the proper instructions. According to other published research, the virtual clinic operation caused confusion and resulted in a high proportion of patients missing their virtual appointments [60, 61]. There may a number of other reasons patients missed their hospital call. For instance, some patients may not be within the network's service region, may only have limited coverage, or may not have had a fully charged phone to receive a call. Technical challenges associated with using such an approach have been documented [62, 63]. Patients' perceptions of and satisfaction with the usage of virtual clinics may also be unfavourable, which may result in them opting to miss future virtual appointments. Indeed, some studies have indicated that this technique of appointments has a low patient satisfaction rating, which results in patients missing the appointment and future appointments [64, 65]. We did not have data regarding virtual clinic appointments for the pre COVID-19 period since this service was only implemented post COVID-19 so we cannot compare or interpret how the implementation of virtual appointments and their attendance rate was affected by the COVID-19 pandemic.

## Limitations

This study has several limitations. First, it is based on a single tertiary referral hospital in the capital city of Muscat, Sultanate of Oman. Hence, the results may be not widely generalisable to other referral hospitals and primary health care settings. Although the aim of this study was to investigate the rates and drivers of missed appointment by clinic and how these may have changed during the COVID-19 pandemic to inform decision makers in the Ministry of Health, future work may need to investigate the impact of missed appointments on patient outcomes. In addition, our study sample included data that covered a period of two years, one-year pre and one year post COVID-19, where other studies looked at the effect of COVID-19 during a short period of time (three month before and three months after). This allowed for a large sample size compared to similar studies. Our research team performed analyses of the results using logistic regression and interaction terms to estimate the association of patient characteristics with missing scheduled appointments compared to similar studies that only reported overall counts and percentages. Although logistic regression was reported to be the most widely utilised approach for binary outcomes, other studies used a variety of statistical models and machine learning models such as Decision Tree, Neural Network and Random Forest [27]. Finally, we used multiple appointments per patients as it was justified by the finding from previous study that the rate of missed appointment across groups of patients with different number of previous appointment was similar. However, other might consider using a model of repeated measure or single appointment per patient [45].

## Conclusions

There was a direct impact of COVID-19 on missed appointments in most clinics. There was an increase in the rate of missed face-to-face and virtual clinics appointments post COVID-19 and a decline in the number of scheduled appointments as well. Substantial increase in the rate of missed appointments was observed during lockdowns period for all clinics. The case mix of patients who missed did not change. Virtual appointments, introduced after start of the pandemic, also had a substantial rate of missed appointments, and cannot be viewed as the single approach to overcome hospital attendance issues. Given the high rates during lockdowns and the ineffectiveness of recently implemented virtual clinics, the results of this study highlight the critical need for strong contingency plans in healthcare systems to ensure uninterrupted access to critical medical services during a crisis.

### Supplementary Information

**Additional file 1: Supplementary Figure 1. **Study sample selection for the face-to-face appointments. **Supplementary Table 1. **Additional baseline characteristics for the study population for each unique patient for all scheduled appointments and stratified by attended and missed face-to-face appointments. **Supplementary Table 2.**  Numbers of scheduled appointments by outpatients clinics at The Royal Hospital stratified by attended and missed face-to-face appointments. **Supplementary Table 3.** Distribution and frequency of face-to-face and virtual clinic appointments during study period. **Supplementary Figure 2.** Rate of missed face-to-face appointments in the top seven clinics pre and post COVID-19. **Supplementary Table 4.** Effects of distance between patient’s residence and The Royal Hospital on missed face-to-face appointments pre and post COVID-19. **Supplementary Table 5.** Predictors of missed hospital face-to-face appointment for the Obstetrics & Gynaecology clinic at The Royal Hospital pre and post COVID-19. **Supplementary Table 6.** Predictors of missed hospital face-to-face appointments for the Paediatrics clinic at The Royal Hospital pre and post COVID-19. **Supplementary Table 7.** Predictors of missed hospital face-to-face appointments for the Diabetics and Endocrine clinic at The Royal Hospital pre and post COVID-19. **Supplementary Table 8.** Predictors of missed hospital face-to-face appointments for the Surgery clinic at The Royal Hospital pre and post COVID-19. **Supplementary Table 9.** Predictors of missed hospital face-to-face appointments for the Oncology clinic at The Royal Hospital pre and post COVID-19. **Supplementary Table 10.** Predictors of missed hospital face-to-face appointments for the Urology clinic at The Royal Hospital pre and post COVID-19. **Supplementary Table 11.** Predictors of missed hospital face-to-face appointments for the Gastroenterology clinic at The Royal Hospital pre and post COVID-19. **Supplementary Table 12.** Characteristics of virtual clinic appointments for each unique patient for all appointments stratified by attended and missed appointments. **Supplementary Table 13.** Distribution of the virtual clinic appointments scheduled within different medical speciality at The Royal Hospital stratified by attended and missed appointments. **Supplementary Table 14.** Effects of distance between patient’s residence and Royal Hospital on missed appointments post COVID-19 face to-face and post COVID-19 virtual clinics.

## Data Availability

The data that support the findings of this study are available from Ministry of Health, Sultanate of Oman, but restrictions apply to the availability of these data, which were used under license for the current study, and so are not publicly available. The corresponding author should be contacted for the process to request data access.

## Abbreviations

ALSHIFA: Health information management system
EHRs: Electronic health records
COVID-19: Coronavirus severe acute respiratory syndrome
WhatsApp: “WhatsApp is a free, multiplatform messaging app that lets you make video and voice calls, send text messages, and more — all with just a Wi-Fi connection
