# Supplementary material for: The impact of the COVID-19 pandemic on rates and predictors of missed hospital appointments in multiple outpatient clinics of The Royal Hospital, Sultanate of Oman: a retrospective study
Source: BMC Health Serv Res. 2023 Dec 19;23:1438. doi: 10.1186/s12913-023-10395-w (PMC10729569; doi:10.1186/s12913-023-10395-w)
Supplement: Supplementary file 1 — Additional file 1: Supplementary Figure 1. Study sample selection for the face-to-face appointments. Supplementary Table 1. Additional baseline characteristics for the study population for each unique patient for all scheduled appointments and stratified by attended and missed face-to-face appointments. Supplementary Table 2. Numbers of scheduled appointments by outpatients clinics at The Royal Hospital stratified by attended and missed face-to-face appointments. Supplementary Table 3. Distribution and frequency of face-to-face and virtual clinic appointments during study period. Supplementary Figure 2. Rate of missed face-to-face appointments in the top seven clinics pre and post COVID-19. Supplementary Table 4. Effects of distance between patient’s residence and The Royal Hospital on missed face-to-face appointments pre and post COVID-19. Supplementary Table 5. Predictors of missed hospital face-to-face appointment for the Obstetrics & Gynaecology clinic at The Royal Hospital pre and post COVID-19. Supplementary Table 6. Predictors of missed hospital face-to-face appointments for the Paediatrics clinic at The Royal Hospital pre and post COVID-19. Supplementary Table 7. Predictors of missed hospital face-to-face appointments for the Diabetics and Endocrine clinic at The Royal Hospital pre and post COVID-19. Supplementary Table 8. Predictors of missed hospital face-to-face appointments for the Surgery clinic at The Royal Hospital pre and post COVID-19. Supplementary Table 9. Predictors of missed hospital face-to-face appointments for the Oncology clinic at The Royal Hospital pre and post COVID-19. Supplementary Table 10. Predictors of missed hospital face-to-face appointments for the Urology clinic at The Royal Hospital pre and post COVID-19. Supplementary Table 11. Predictors of missed hospital face-to-face appointments for the Gastroenterology clinic at The Royal Hospital pre and post COVID-19. Supplementary Table 12. Characteristics of virtual clinic appointments [file 12913_2023_10395_MOESM1_ESM.docx]

**A summary of the COVID-19 pandemic in Oman**

In the beginning of the pandemic, the government implemented a national lockdown and restriction of movement.^1^’^2^’^3^’^4^ Patients with medical appointments were given permission to travel between cities to attend their appointments where they had to show their appointment slips (SMS reminder message) in the checkpoints between cities and governorates. As the number of cases increased, more measures to control the spread of the virus were implemented.^5^ Non–urgent appointments were cancelled and rescheduled during the peak time of the pandemic and only urgent appointments were kept for patients with serious conditions or in need of urgent medical care. The Royal Hospital provided medical services for COVID-19 patients during the pandemic period. Meanwhile, temporary hospital was opened in May2020 to provide care to COVID-19 patients as the number of admission and ICU occupancy increased as cases increased along deaths^6^. Different waves of the pandemic were reported as the number of daily cases went up. The total number of confirmed COVID-19 cases was 159,218 and total death was 1,678 by March 31^st^, 2021.^7^

**Reference:**

1. Oman T of. Lockdown of all governorates in Oman to begin from Saturday. *Times of Oman*. https://timesofoman.com/article/3017354/oman/government/lockdown-of-all-governorates-in-oman-to-begin-from-saturday-at-7pm. Published 2020.

2. OBSERVER O. Muscat lockdown lifted muttrah still in isolation. *OMAN OBSERVER*. https://www.omanobserver.om/article/13015/CORONAVIRUS/muscat-lockdown-lifted-muttrah-still-in-isolation. Published May 27, 2020.

3. Daily M. Lockdown begins in Dhofar, Jebel Shams, Jebel Akhdar, Masirah and Duqm.https://www.muscatdaily.com/2020/06/13/lockdown-begins-in-dhofar-jebel-shams-jebel-akhdar-masirah-and-duqm/. Published June 13, 2020.

4. MANSOOR Z. Oman extends lockdown of Muscat; bans gatherings during Ramadan The security measures and checkpoints that have b. *Gulf Business*. https://gulfbusiness.com/oman-extends-lockdown-of-muscat-bans-gatherings-during-ramadan/. Published April 22, 2020.

5. Wahaibi A Al, Maani A Al, Alyaquobi F, et al. The impact of mobility restriction strategies in the control of the COVID-19 pandemic: Modelling the relation between COVID-19 health and community mobility data. *Int J Environ Res Public Health*. 2021;18(19). doi:10.3390/ijerph181910560

6. Oman T of. Temporary OMA Hospital for COVID-19 opens in Oman.https://timesofoman.com/article/88198-temporary-oma-hospital-for-covid-19-opens-in-oman?msclkid=b4b5d7b0b01d11ecb29071dba277204a. Published May 17, 2020.

7. Worldmeters. worldometers.info. https://www.worldometers.info/coronavirus/country/oman/. Published 2021. Accessed August 20, 2021.

| 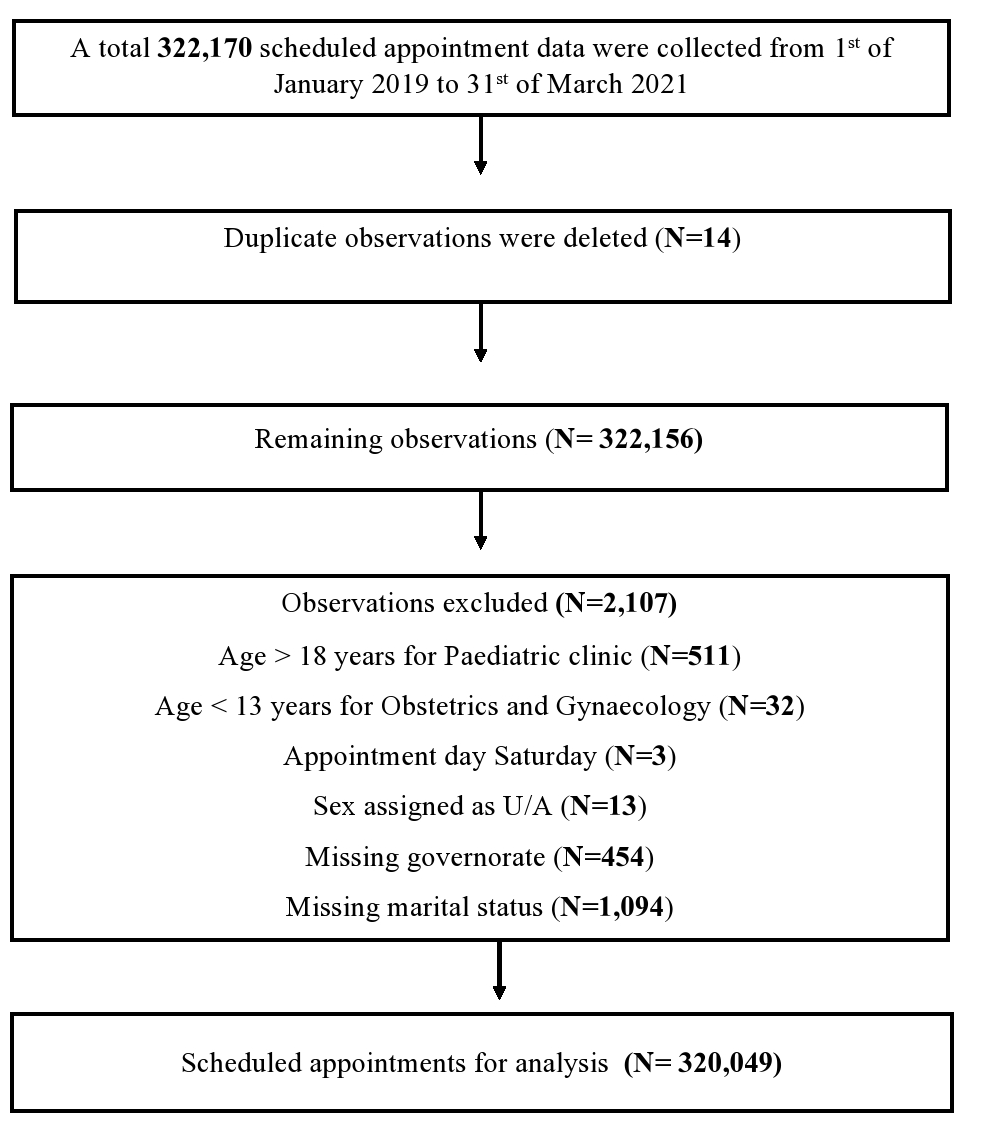 |
| --- |
| **Supplementary Figure 1: Study sample selection for the face-to-face appointments** |

| **Supplementary Table 1: Additional baseline characteristics for the study population for each unique patient for all scheduled appointments and stratified by attended and missed face-to-face appointments** | | | | | |
| --- | --- | --- | --- | --- | --- |
| **Variable** | **Characteristics of Patients**  **N=80835** | **Characteristics of Appointments N=320049** | **Attended N (%)**  **256792 (80.2)** | **Missed N (%)**  **63257 (19.8)** | |
| **Appointment month** | | | ***p value <0.001** | | |
| January | 11245 (13.9) | 41594 (13.0) | 34872(83.8) | | 6722 (16.2) |
| February | 10385 (12.8) | 37636 (11.8) | 32145(85.4) | | 5491 (14.6) |
| March | 11281 (14.0) | 41183 (12.9) | 34005 (82.6) | | 7178 (17.4) |
| April | 5827 (7.2) | 25317 (7.9) | 18185 (71.8) | | 7132 (28.2) |
| May | 4735 (5.9) | 20002 (6.2) | 14739 (73.7) | | 5263 (26.3) |
| June | 4994 (6.2) | 22147 (6.9) | 16582 (74.9) | | 5565 (25.1) |
| July | 5247 (6.5) | 22073 (6.9) | 17165 (77.8) | | 4908 (22.2) |
| August | 3956 (4.9) | 16756 (5.2) | 12803 (76.4) | | 3953 (23.6) |
| September | 5775 (7.1) | 24259 (7.6) | 19392 (79.9) | | 4867 (20.1) |
| October | 5878 (7.3) | 24234 (7.6) | 19607 (80.9) | | 4627 (19.1) |
| November | 4908 (6.1) | 19264 (6.0) | 15603 (81.0) | | 3661 (19.0) |
| December | 6604 (8.2) | 25584 (8.0) | 21694 (84.8) | | 3890 (15.2) |
| **Appointment year** | | | ***p value <0.001** | | |
| 2019 | 40219 (49.8) | 151726 47.4) | 126142(83.1) | 25584 (16.9) | |
| 2020 | 28928 (35.8) | 128861(40.3) | 96857 (75.2) | 32004 (24.8) | |
| 2021 † | 11688 (14.5) | 39462 (12.3) | 33793 (85.6) | 5669 (14.4) | |
| **Marital status** | | | ***p value <0.001** | | |
| Child (<13 years) | 20340 (25.2) | 70574 (22.1) | 54766 (77.6) | 15808 (22.4) | |
| Single | 15259 (18.9) | 62265 (19.5) | 48416 (77.8) | 13849 (22.2) | |
| Married | 44421 (55.0) | 183828 (57.4) | 150940 (82.1) | 32888 (17.9) | |
| Divorced | 336 (0.4) | 1398 (0.4) | 1109 (79.3) | 289 (20.7) | |
| Widow | 479 (0.6) | 1984 (0.6) | 1561 (78.7) | 423 (21.3) | |
| **Nationality** | | | ***p value<0.001** | | |
| Omani | 77659(96.1) | 308223 (96.3) | 247141 (80.2) | 61082 (19.8) | |
| Non-Omani | 3176 (3.9) | 11826 (3.7) | 9651 (81.6) | 2175 (18.4) | |
| *p value calculated using the Tableone R package which summarises categorical and continuous variables. The chi-squared test was used to calculate the p value for categorical variable and one-way analysis of variance (ANOVA) for continuous variables.)  † The data for 2021 included only three months from (1/1/2021 to 31/3/2021). Between 1^st^ Jan 2019 – 31^st^ March 2021, for regression analysis 11,516 observations were removed from the between covid-19 period | | | | | |

| **Supplementary Table 2: Numbers of scheduled appointments by outpatients clinics at The Royal Hospital stratified by attended and missed face-to-face appointments** | | | | | |
| --- | --- | --- | --- | --- | --- |
|  | **By**  **Patients**  **(N)** | **By Appointment**  **(N)** | | **Attended**  **N (%)** | **Missed**  **N (%)** |
|  | 80835 | 320049 | | 256792 (80.2) | 63257 (19.8) |
| **Clinic** | | | | ***p value <0.001** | |
| Acute Medical Admission | 175 (0.2) | 427 (0.1) | 374 (87.6) | | 53 (12.4) |
| Adolescent Medicine | 14 (0.0) | 89 (0.0) | 71 (79.8) | | 18 (20.2) |
| Anaesthesia | 1669 (2.1) | 5793 (1.8) | 4872 (84.1) | | 921 (15.9) |
| Cardiology | 8725 (10.8) | 23643 (7.4) | 18635 (78.8) | | 5008 (21.2) |
| Chest Medicine | 1583 (2.0) | 6343 (2.0) | 5129 (80.9) | | 1214 (19.1) |
| Clinical Haematology | 1285 (1.6) | 7010 (2.2) | 5718 (81.6) | | 1292 (18.4) |
| Clinical Immunology & Allergy | 331 (0.4) | 765 (0.2) | 613 (80.1) | | 152 (19.9) |
| Clinical Radiology | 14 (0.0) | 54 (0.0) | 53 (98.1) | | 1 (1.9) |
| Dental | 356 (0.4) | 2305 (0.7) | 1656 (71.8) | | 649 (28.2) |
| Diabetes & Endocrine | 4777 (5.9) | 43830 (13.7) | 34290 (78.2) | | 9540 (21.8) |
| Ear Nose Throat | 13 (0.0) | 37 (0.0) | 34 (91.9) | | 3 (8.1) |
| Gastroenterology | 2421 (3.0) | 9471 (3.0) | 6843 (72.3) | | 2628 (27.7) |
| Genetic Disorders | 142 (0.2) | 478 (0.1) | 320 (66.9) | | 158 (33.1) |
| Infectious Diseases | 453 (0.6) | 3860 (1.2) | 2459 (63.7) | | 1401 (36.3) |
| Endocrinology | 624 (0.8) | 2570 (0.8) | 2019 (78.6) | | 551 (21.4) |
| Hyperbaric Oxygen Therapy | 45 (0.1) | 182 (0.1) | 167 (91.8) | | 15 (8.2) |
| Nephrology | 954 (1.2) | 6512 (2.0) | 5131 (78.8) | | 1381 (21.2) |
| Obstetrics & Gynaecology | 16248(20.1) | 53836 (16.8) | 49269 (91.5) | | 4567 (8.5) |
| Oncology | 3251 (4.0) | 21594 (6.7) | 18258 (84.6) | | 3336 (15.4) |
| Paediatric | 22603(28.0) | 82611 (25.8) | 63537 (76.9) | | 19074 (23.1) |
| Psychiatry | 31 (0.0) | 203 (0.1) | 191 (94.1) | | 12 (5.9) |
| Rheumatology | 2238 (2.8) | 11325 (3.5) | 7585 (67.0) | | 3740 (33.0) |
| Surgery | 7392 (9.1) | 23616 (7.4) | 18698 (79.2) | | 4918 (20.8) |
| Urology | 5091 (6.3) | 12030 (3.8) | 9591 (79.7) | | 2439 (20.3) |
| ¥VIP and Hospital staff | 400 (0.5) | 1465 (0.5) | 1279 (87.3) | | 186 (12.7) |
| ¥Very important person  *p value calculated using the Tableone R package which summarises categorical and continuous variables. The chi-squared test was used to calculate the p value for categorical variable and one-way analysis of variance (ANOVA) for continuous variables. Between 1^st^ Jan 2019 – 31^st^ March 2021, for regression analysis 11,516 observations were removed from the between covid-19 period | | | | | |

| **Supplementary Table 3: Distribution and frequency of face-to-face and virtual clinic appointments during study period** | | | | | | | | | |
| --- | --- | --- | --- | --- | --- | --- | --- | --- | --- |
|  | **Face-to-Face appointments** | | | | *** Virtual clinic appointments** | | | | |
| **Week Number** | Date | Attended | Missed | Total | % Missed | Attended | Missed | Total | % Missed |
| 1 | 30/12/2018 | 1563 | 250 | 1813 | 13.8 |  | | | |
| 2 | 06/01/2019 | 2806 | 348 | 3154 | 11.0 |  | | | |
| 3 | 13/01/2019 | 2695 | 397 | 3092 | 12.8 |  | | | |
| 4 | 20/01/2019 | 2632 | 428 | 3060 | 14.0 |  | | | |
| 5 | 27/01/2019 | 2327 | 411 | 2738 | 15.0 |  | | | |
| 6 | 03/02/2019 | 2365 | 515 | 2880 | 17.9 |  | | | |
| 7 | 10/02/2019 | 2533 | 467 | 3000 | 15.6 |  | | | |
| 8 | 17/02/2019 | 2541 | 449 | 2990 | 15.0 |  | | | |
| 9 | 24/02/2019 | 2704 | 470 | 3174 | 14.8 |  | | | |
| 10 | 03/03/2019 | 2723 | 602 | 3325 | 18.1 |  | | | |
| 11 | 10/03/2019 | 3101 | 580 | 3681 | 15.8 |  | | | |
| 12 | 17/03/2019 | 2961 | 587 | 3548 | 16.5 |  | | | |
| 13 | 24/03/2019 | 2833 | 664 | 3497 | 19.0 |  | | | |
| 14 | 31/03/2019 | 2088 | 745 | 2833 | 26.3 |  | | | |
| 15 | 07/04/2019 | 2787 | 787 | 3574 | 22.0 |  | | | |
| 16 | 14/04/2019 | 2734 | 643 | 3377 | 19.0 |  | | | |
| 17 | 21/04/2019 | 2812 | 564 | 3376 | 16.7 |  | | | |
| 18 | 28/04/2019 | 2847 | 582 | 3429 | 17.0 |  | | | |
| 19 | 05/05/2019 | 2496 | 769 | 3265 | 23.6 |  | | | |
| 20 | 12/05/2019 | 2310 | 678 | 2988 | 22.7 |  | | | |
| 21 | 19/05/2019 | 2260 | 688 | 2948 | 23.3 |  | | | |
| 22 | 26/05/2019 | 2162 | 551 | 2713 | 20.3 |  | | | |
| 23 | 02/06/2019 | 428 | 114 | 542 | 21.0 |  | | | |
| 24 | 09/06/2019 | 2387 | 550 | 2937 | 18.7 |  | | | |
| 25 | 16/06/2019 | 2567 | 637 | 3204 | 19.9 |  | | | |
| 26 | 23/06/2019 | 2578 | 526 | 3104 | 16.9 |  | | | |
| 27 | 30/06/2019 | 2452 | 472 | 2924 | 16.1 |  | | | |
| 28 | 07/07/2019 | 2406 | 519 | 2925 | 17.7 |  | | | |
| 29 | 14/07/2019 | 2401 | 505 | 2906 | 17.4 |  | | | |
| 30 | 21/07/2019 | 1850 | 459 | 2309 | 19.9 |  | | | |
| 31 | 28/07/2019 | 2302 | 551 | 2853 | 19.3 |  | | | |
| 32 | 04/08/2019 | 2271 | 581 | 2852 | 20.4 |  | | | |
| **33 | 11/08/2019 | 0 | 0 | 0 | 0.0 |  | | | |
| 34 | 18/08/2019 | 2378 | 529 | 2907 | 18.2 |  | | | |
| 35 | 25/08/2019 | 2378 | 554 | 2932 | 18.9 |  | | | |
| 36 | 01/09/2019 | 2042 | 417 | 2459 | 17.0 |  | | | |
| 37 | 08/09/2019 | 2716 | 531 | 3247 | 16.4 |  | | | |
| 38 | 15/09/2019 | 2670 | 492 | 3162 | 15.6 |  | | | |
| 39 | 22/09/2019 | 2700 | 499 | 3199 | 15.6 |  | | | |
| 40 | 29/09/2019 | 2589 | 532 | 3121 | 17.0 |  | | | |
| 41 | 06/10/2019 | 2653 | 468 | 3121 | 15.0 |  | | | |
| 42 | 13/10/2019 | 2569 | 401 | 2970 | 13.5 |  | | | |
| 43 | 20/10/2019 | 2743 | 439 | 3182 | 13.8 |  | | | |
| 44 | 27/10/2019 | 2485 | 476 | 2961 | 16.1 |  | | | |
| 45 | 03/11/2019 | 2702 | 479 | 3181 | 15.1 |  | | | |
| 46 | 10/11/2019 | 1995 | 347 | 2342 | 14.8 |  | | | |
| 47 | 17/11/2019 | 2688 | 429 | 3117 | 13.8 |  | | | |
| 48 | 24/11/2019 | 897 | 121 | 1018 | 11.9 |  | | | |
| 49 | 01/12/2019 | 2724 | 436 | 3160 | 13.8 |  | | | |
| 50 | 08/12/2019 | 2618 | 413 | 3031 | 13.6 |  | | | |
| 51 | 15/12/2019 | 2709 | 388 | 3097 | 12.5 |  | | | |
| 52 | 22/12/2019 | 2499 | 340 | 2839 | 12.0 |  | | | |
| 53 | 29/12/2019 | 2534 | 375 | 2909 | 12.9 |  | | | |
| 54 | 05/01/2020 | 2666 | 384 | 3050 | 12.6 |  | | | |
| ***55 | 12/01/2020 | 2229 | 1491 | 3720 | 40.1 |  | | | |
| 56 | 19/01/2020 | 2748 | 442 | 3190 | 13.9 |  | | | |
| 57 | 26/01/2020 | 2697 | 432 | 3129 | 13.8 |  | | | |
| 58 | 02/02/2020 | 2650 | 448 | 3098 | 14.5 |  | | | |
| 59 | 09/02/2020 | 2786 | 429 | 3215 | 13.3 |  | | | |
| 60 | 16/02/2020 | 2804 | 369 | 3173 | 11.6 |  | | | |
| 61 | 23/02/2020 | 2902 | 406 | 3308 | 12.3 |  | | | |
| 62 | 01/03/2020 | 2901 | 454 | 3355 | 13.5 |  | | | |
| 63 | 08/03/2020 | 2947 | 446 | 3393 | 13.1 |  | | | |
| 64 | 15/03/2020 | 2372 | 660 | 3032 | 21.8 |  | | | |
| 65 | 22/03/2020 | 1091 | 645 | 1736 | 37.2 |  | | | |
| 66 | 29/03/2020 | 1597 | 1053 | 2650 | 39.7 |  | | | |
| 67 | 05/04/2020 | 1550 | 1016 | 2566 | 39.6 |  | | | |
| 68 | 12/04/2020 | 1537 | 997 | 2534 | 39.3 | 0 | 1 | 1 | 100.0 |
| 69 | 19/04/2020 | 1563 | 1022 | 2585 | 39.5 | 22 | 9 | 31 | 29.0 |
| 70 | 26/04/2020 | 1314 | 746 | 2060 | 36.2 | 50 | 5 | 55 | 9.1 |
| 71 | 03/05/2020 | 1344 | 781 | 2125 | 36.8 | 72 | 25 | 97 | 25.8 |
| 72 | 10/05/2020 | 1366 | 691 | 2057 | 33.6 | 91 | 22 | 113 | 19.5 |
| 73 | 17/05/2020 | 1293 | 704 | 1997 | 35.3 | 75 | 14 | 89 | 15.7 |
| 74 | 24/05/2020 | 83 | 11 | 94 | 11.7 | 1 | 4 | 5 | 80.0 |
| 75 | 31/05/2020 | 1910 | 846 | 2756 | 30.7 | 311 | 114 | 425 | 26.8 |
| 76 | 07/06/2020 | 1883 | 892 | 2775 | 32.1 | 355 | 130 | 485 | 26.8 |
| 77 | 14/06/2020 | 1845 | 755 | 2600 | 29.0 | 385 | 158 | 543 | 29.1 |
| 78 | 21/06/2020 | 1753 | 791 | 2544 | 31.1 | 338 | 179 | 517 | 34.6 |
| 79 | 28/06/2020 | 1698 | 747 | 2445 | 30.6 | 362 | 163 | 525 | 31.0 |
| 80 | 05/07/2020 | 1664 | 619 | 2283 | 27.1 | 349 | 137 | 486 | 28.2 |
| 81 | 12/07/2020 | 1599 | 556 | 2155 | 25.8 | 291 | 92 | 383 | 24.0 |
| 82 | 19/07/2020 | 1484 | 536 | 2020 | 26.5 | 278 | 108 | 386 | 28.0 |
| 83 | 26/07/2020 | 1314 | 632 | 1946 | 32.5 | 260 | 155 | 415 | 37.3 |
| 84 | 02/08/2020 | 72 | 171 | 243 | 70.4 | 6 | 52 | 58 | 89.7 |
| 85 | 09/08/2020 | 1689 | 651 | 2340 | 27.8 | 261 | 191 | 452 | 42.3 |
| 86 | 16/08/2020 | 1499 | 595 | 2094 | 28.4 | 275 | 142 | 417 | 34.1 |
| 87 | 23/08/2020 | 1432 | 545 | 1977 | 27.6 | 235 | 114 | 349 | 32.7 |
| 88 | 30/08/2020 | 1782 | 624 | 2406 | 25.9 | 311 | 166 | 477 | 34.8 |
| 89 | 06/09/2020 | 1880 | 576 | 2456 | 23.5 | 252 | 176 | 428 | 41.1 |
| 90 | 13/09/2020 | 1968 | 581 | 2549 | 22.8 | 329 | 162 | 491 | 33.0 |
| 91 | 20/09/2020 | 1933 | 569 | 2502 | 22.7 | 320 | 183 | 503 | 36.4 |
| 92 | 27/09/2020 | 1470 | 826 | 2296 | 36.0 | 252 | 224 | 476 | 47.1 |
| 93 | 04/10/2020 | 1873 | 574 | 2447 | 23.5 | 345 | 156 | 501 | 31.1 |
| 94 | 11/10/2020 | 1916 | 556 | 2472 | 22.5 | 376 | 160 | 536 | 29.9 |
| 95 | 18/10/2020 | 2021 | 634 | 2655 | 23.9 | 364 | 185 | 549 | 33.7 |
| 96 | 25/10/2020 | 1663 | 536 | 2199 | 24.4 | 329 | 136 | 465 | 29.2 |
| 97 | 01/11/2020 | 1845 | 525 | 2370 | 22.2 | 282 | 243 | 525 | 46.3 |
| 98 | 08/11/2020 | 2043 | 553 | 2596 | 21.3 | 383 | 222 | 605 | 36.7 |
| 99 | 15/11/2020 | 1678 | 521 | 2199 | 23.7 | 279 | 138 | 417 | 33.1 |
| 100 | 22/11/2020 | 869 | 468 | 1337 | 35.0 | 117 | 98 | 215 | 45.6 |
| 101 | 29/11/2020 | 2145 | 555 | 2700 | 20.6 | 401 | 167 | 568 | 29.4 |
| 102 | 06/12/2020 | 2191 | 453 | 2644 | 17.1 | 433 | 167 | 600 | 27.8 |
| 103 | 13/12/2020 | 2182 | 510 | 2692 | 18.9 | 407 | 211 | 618 | 34.1 |
| 104 | 20/12/2020 | 2076 | 399 | 2475 | 16.1 | 372 | 159 | 531 | 29.9 |
| 105 | 27/12/2020 | 1971 | 410 | 2381 | 17.2 | 367 | 189 | 556 | 34.0 |
| 106 | 03/01/2021 | 2734 | 465 | 3199 | 14.5 | 382 | 177 | 559 | 31.7 |
| 107 | 10/01/2021 | 2753 | 405 | 3158 | 12.8 | 383 | 152 | 535 | 28.4 |
| 108 | 17/01/2021 | 2669 | 510 | 3179 | 16.0 | 423 | 184 | 607 | 30.3 |
| 109 | 24/01/2021 | 2764 | 503 | 3267 | 15.4 | 471 | 177 | 648 | 27.3 |
| 110 | 31/01/2021 | 2667 | 481 | 3148 | 15.3 | 440 | 161 | 601 | 26.8 |
| 111 | 07/02/2021 | 2694 | 488 | 3182 | 15.3 | 421 | 209 | 630 | 33.2 |
| 112 | 14/02/2021 | 2677 | 490 | 3167 | 15.5 | 465 | 194 | 659 | 29.4 |
| 113 | 21/02/2021 | 2776 | 457 | 3233 | 14.1 | 442 | 215 | 657 | 32.7 |
| 114 | 28/02/2021 | 2678 | 520 | 3198 | 16.3 | 451 | 225 | 676 | 33.3 |
| 115 | 07/03/2021 | 2058 | 280 | 2338 | 12.0 | 361 | 137 | 498 | 27.5 |
| 116 | 14/03/2021 | 2558 | 364 | 2922 | 12.5 | 479 | 241 | 720 | 33.5 |
| 117 | 21/03/2021 | 2748 | 399 | 3147 | 12.7 | 544 | 223 | 767 | 29.1 |
| 118 | 28/03/2021 | 2017 | 307 | 2324 | 13.2 | 455 | 195 | 650 | 30.0 |
|  |  | 256792 | 63257 | 320049 |  | 15653 | 7447 | 23100 |  |
| **Lockdown period 1** (from Week 66 to Week 75, 1/4/2020 to 29/5/2020)  **Lockdown period 2** (from Week77 to Week 79, 13/6/2020 to 3/7/2020)  **Lockdown period 3** (from Week 83 to Week 84, 25/7/2020 to 8/8/2020)  *****The virtual clinic appointments started in 16/4/2020  ** One week public holiday (with no scheduled appointments)  *** Three days of mourning following the death of Sultan Qaboos bin Said (two days of scheduled appointments only). | | | | | | | | | |


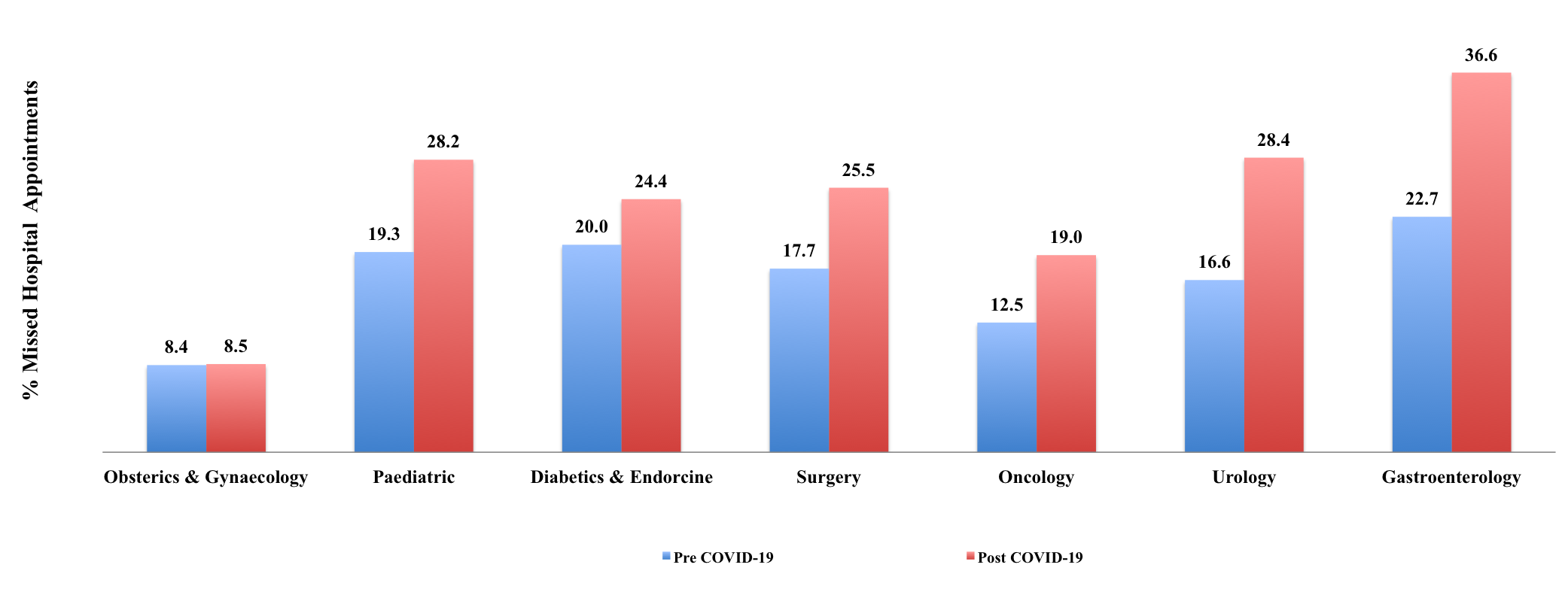


|  |
| --- |
| **Supplementary Figure 2: Rate of missed face-to-face appointments in the top seven clinics pre and post COVID-19** Pre COVID-19 period from (1/1/2019 to 23/2/2020) and Post COVID-19 from (1/4/2020 to 31/3/2021) |

| **Supplementary Table 4: Effects of distance between patient’s residence and The Royal Hospital on missed face-to-face appointments pre and post COVID-19** | | | | |
| --- | --- | --- | --- | --- |
| **Governorate** | **Minimum-Maximum Distance to The Royal Hospital (Km)** | **Odd Ratio (95% CI)** | | |
|  |  | **Pre**  **COVID-19** | **Post**  **COVID-19** | **^ Interaction** |
| Muscat | 1-90 | **Reference** | | |
| South Batina | 50-165 | 1.23 (1.06-1.41)* | 1.37 (1.16-1.61)* | 1.12(0.91-1.39) |
| AL Dhakiliya | 70-206 | 1.19 (0.98-1.43) | 1.50 (1.18-1.90)* | 1.20 (0.89-1.62) |
| North Batina | 114-262 | 1.18 (1.02-1.36)* | 1.07 (0.92-1.24) | 0.85 (0.70-1.04) |
| North Sharqiya | 173-290 | 1.00 (0.94-1.06) | 1.01 (0.95-1.08) | 1.02 (0.94-1.11) |
| South Sharqiya | 205-320 | 1.10 (1.04-1.16)* | 1.06 (1.00-1.12)* | 0.93 (0.86-1.01) |
| AL Dhahira | 245-340 | 1.03 (0.97-1.09) | 0.98 (0.92-1.04) | 0.98 (0.91-1.07) |
| AL Buriami | 285-350 | 1.23 (1.15-1.32)* | 1.21 (1.13-1.30)* | 1.02 (0.93-1.13) |
| AL Wusta | 365-490 | 1.11 (1.04-1.20)* | 1.08 (1.00-1.16)* | 0.97 (0.88-1.08) |
| Musandam | 540-620 | 1.16 (1.07-1.26)* | 1.03 (0.94-1.12) | 0.86 (0.76-0.97) |
| Dhofar | 800-1120 | 1.15 (0.90-1.48) | 1.01 (0.75-1.35) | 0.97 (0.66-1.41) |
| ^ Interaction: models adjusted with an interaction term for Pre and Post COVID-19  *Statistically significant | | | | |

| **Supplementary Table 5: Predictors of missed hospital face-to-face appointment for the Obstetrics & Gynaecology clinic at The Royal Hospital pre and post COVID-19** | | |
| --- | --- | --- |
|  | **Pre COVID-19 (N=29430)** | **Post COVID-19**  **(N=22420)** |
|  | **OR (95% CI)** | **OR (95% CI)** |
| **Age category** |  |  |
| 14-18 years old | 1.31 (0.78-2.20) | 1.38 (0.76-2.51) |
| 19-30 years old | 1.01(0.91-1.12) | 0.84 (0.75-0.95)* |
| 31-40 years old | **Reference** | **Reference** |
| 41-50 years old | 1.40 (1.25-1.57)* | 1.12 (0.98-1.28) |
| 51-60 years old | 1.44 (1.18-1.77)* | 1.33 (1.03-1.71)* |
| 61-70 years old | 1.93 (1.40-2.67)* | 1.12 (0.74-1.68) |
| 71-80 years old | 1.59 (0.93-2.70) | 2.02 (1.17-3.47)* |
| > 80 years old | 1.44 (0.48-4.30) | 0.97 (0.26-3.59) |
| **Appointment day** |  |  |
| Sunday | **Reference** | **Reference** |
| Monday | 1.07 (0.92-1.23) | 1.02 (0.87-1.19) |
| Tuesday | 1.40 (1.22-1.61)* | 1.24 (1.06-1.44)* |
| Wednesday | 1.04 (0.90-1.21) | 1.24 (1.06-1.45)* |
| Thursday | 1.04 (0.90-1.20) | 1.01 (0.85-1.19) |
| **Service cost** |  |  |
| Pay visit and registration fees | **Reference** | **Reference** |
| Pay all medical service fees | 0.86 (0.50-1.49) | 0.62 (0.33-1.19) |
| Under social affair coverage** | 0.73 (0.52-1.01) | 1.24 (0.88-1.74) |
| **Appointment waiting days** |  |  |
| ≤ 30 days | **Reference** | **Reference** |
| 31- 60 days | 1.34 (1.19-1.51)* | 1.52 (1.33-1.73)* |
| 61-90 days | 2.53 (2.17-2.95)* | 2.68 (2.27-3.17)* |
| 91- 120 days | 3.61 (3.09-4.23)* | 3.55 (2.94-4.29)* |
| > 120 days | 5.10 (4.53-5.74)* | 6.23 (5.42-7.16)* |
| **Marital status** |  |  |
| Single | 1.19 (1.05-1.34)* | 1.12 (0.98-1.29) |
| Married | **Reference** | **Reference** |
| Divorced | 1.46 (0.82-2.60) | 1.51 (0.76-3.02) |
| Widow | 0.79 (0.33-1.92) | 1.17 (0.49-2.80) |
| **Nationality** |  |  |
| Omani | **Reference** | **Reference** |
| Non-Omani | 1.30 (0.75-2.26) | 1.21 (0.63-2.31) |
| **Exempted from all medical service fees  *Statistically significant | | |

| **Supplementary Table 6: Predictors of missed hospital face-to-face appointments for the Paediatrics clinic at The Royal Hospital pre and post COVID-19** | | |  |
| --- | --- | --- | --- |
|  | **Pre COVID-19 (N=46026)** | **Post COVID-19**  **(N=33418)** | |
|  | **OR (95% CI)** | **OR (95% CI)** | |
| **Sex** |  |  | |
| Female | **Reference** | | |
| Male | 0.89 (0.85-0.93)* | 0.91 (0.87-0.96)* | |
| **Age category** |  |  | |
| ≤ 5 years old | 1.31 (1.21-1.42)* | 1.20 (1.11-1.31)* | |
| 6-10 years old | 1.06 (0.99-1.14) | 1.09 (1.02-1.17)* | |
| 11-13 years old | **Reference** | | |
| 14-18 years old | 1.38 (1.28-1.49)* | 1.30 (1.20-1.41)* | |
| **Appointment day** |  |  | |
| Sunday | **Reference** | | |
| Monday | 0.98 (0.91-1.05) | 0.90 (0.84-0.97)* | |
| Tuesday | 0.93 (0.86-1.00) | 1.15 (1.07-1.24)* | |
| Wednesday | 0.94 (0.87-1.01) | 1.16 (1.07-1.25)* | |
| Thursday | 1.02 (0.95-1.10) | 1.08 (1.00-1.17)* | |
| **Service cost** |  |  | |
| Pay visit and registration fees | 0.95 (0.89-1.02) | 0.88 (0.81-0.95) | |
| Pay all medical service fees | 0.73 (0.52-1.03) | 1.05 (0.73-1.51) | |
| ≤ 2 years old* | **Reference** | | |
| Under Social Affair coverage** | 1.06 (0.97-1.16) | 1.00 (0.91-1.10) | |
| **Appointment waiting days** |  |  | |
| ≤ 30 days | **Reference** | | |
| 31- 60 days | 0.90 (0.83-0.98)* | 1.16 (1.04-1.28)* | |
| 61-90 days | 0.93 (0.85-1.01) | 1.33 (1.21-1.46)* | |
| 91- 120 days | 0.92 (0.85-0.99)* | 1.36 (1.25-1.47)* | |
| > 120 days | 0.81 (0.77-0.87)* | 1.29 (1.22-1.37)* | |
| **Nationality** |  |  | |
| Omani | **Reference** | | |
| Non-Omani | 1.27 (0.89-1.81) | 0.91 (0.62-1.33) | |
| **Exempted from all medical service fees  *Statistically significant | | | |

| **Supplementary Table 7: Predictors of missed hospital face-to-face appointments for the Diabetics and Endocrine clinic at The Royal Hospital pre and post COVID-19** | | |
| --- | --- | --- |
|  | **Pre COVID-19 (N=24589)** | **Post COVID-19**  **(N=17649)** |
|  | **OR (95% CI)** | **OR (95% CI)** |
| **Sex** |  |  |
| Female | **Reference** | |
| Male | 1.09 (1.02-1.18)* | 0.92 (0.85-1.01) |
| **Age category** |  |  |
| 19-30 years old | 1.00 (0.92-1.10) | 0.97 (0.88-1.07) |
| 31-40 years old | **Reference** | |
| 41-50 years old | 1.13 (1.04-1.23)* | 0.96 (0.87-1.05) |
| 51-60 years old | 1.12 (1.00-1.25)* | 0.96 (0.85-1.10) |
| 61-70 years old | 0.89 (0.76-1.03) | 0.81 (0.70-0.95)* |
| 71-80 years old | 0.83 (0.63-1.10) | 0.75 (0.56-0.99)* |
| > 80 years old | 1.08 (0.64-1.84) | 0.52 (0.24-1.11) |
| **Appointment day** |  |  |
| Sunday | **Reference** | |
| Monday | 0.91 (0.82-1.00) | 0.89 (0.80-1.00) |
| Tuesday | 1.03 (0.93-1.13) | 0.96 (0.87-1.07) |
| Wednesday | 1.08 (0.98-1.19) | 1.10 (0.99-1.23) |
| Thursday | 0.72 (0.64-0.80)* | 0.62 (0.55-0.70)* |
| **Service cost** |  |  |
| Pay visit and registration fees | **Reference** | |
| Pay all medical service fees | 0.82 (0.56-1.20) | 0.55 (0.38-0.80) |
| Under social affair coverage** | 0.97 (0.84-1.13) | 1.24 (1.05-1.47)* |
| **Appointment waiting days** |  |  |
| ≤ 30 days | **Reference** | |
| 31-60 days | 1.70 (1.55-1.87)* | 1.18 (1.04-1.33)* |
| 61-90 days | 1.48 (1.32-1.66)* | 1.45 (1.25-1.68)* |
| 91-120 days | 1.57 (1.41-1.74)* | 1.60 (1.39-1.84)* |
| > 120 days | 1.32 (1.19-1.48)* | 1.60 (1.42-1.80)* |
| **Marital status** |  |  |
| Single | 1.08 (1.00-1.17)***** | 0.95 (0.86-1.04) |
| Married | **Reference** | |
| Divorced | 0.97 (0.67-1.42) | 0.84 (0.51-1.40) |
| Widow | 1.66 (1.15-2.39)* | 0.72 (0.37-1.37) |
| **Nationality** |  |  |
| Omani | **Reference** | |
| Non-Omani | 1.18 (0.79-1.78) | 1.50 (1.03-2.17)* |
| **Exempted from all medical service fees  *Statistically significant | | |

| **Supplementary Table 8: Predictors of missed hospital face-to-face appointments for the Surgery clinic at The Royal Hospital pre and post COVID-19** | | |  |
| --- | --- | --- | --- |
|  | **Pre COVID-19 (N=13726)** | **Post COVID-19**  **(N=9055)** | |
|  | **OR (95% CI)** | **OR (95% CI)** | |
| **Sex** |  |  | |
| Female | **Reference** | | |
| Male | 0.99 (0.90-1.09) | 1.00 (0.90-1.11) | |
| **Age category** |  |  | |
| 19-30 years old | 0.94 (0.82-1.09) | 0.91 (0.77-1.09) | |
| 31-40 years old | **Reference** | | |
| 41-50 years old | 1.00 (0.88-1.14) | 1.01 (0.88-1.17) | |
| 51-60 years old | 0.97 (0.84-1.12) | 0.93 (0.79-1.09) | |
| 61-70 years old | 0.96 (0.81-1.13) | 0.91 (0.76-1.08) | |
| 71-80 years old | 0.97(0.79-1.19) | 0.89 (0.71-1.10) | |
| > 80 years old | 0.72 (0.45-1.17) | 0.80 (0.54-1.18) | |
| **Appointment day** |  |  | |
| Sunday | **Reference** | | |
| Monday | 0.98 (0.85-1.14) | 1.03 (0.87-1.22) | |
| Tuesday | 1.17 (1.01-1.36)* | 0.96 (0.82-1.14) | |
| Wednesday | 1.04 (0.90-1.20) | 1.20 (1.02-1.41)* | |
| Thursday | 1. 33 (1.14-1.55)* | 1.52 (1.26-1.84)* | |
| **Service cost** |  |  | |
| Pay visit and registration fees | **Reference** | | |
| Pay all medical service fees | 0.76 (0.43-1.33) | 1.02 (0.52-1.98) | |
| Under social affair coverage** | 1.04 (0.86-1.27) | 1.11 (0.91-1.36) | |
| **Appointment waiting days** |  |  | |
| ≤ 30 days | **Reference** | | |
| 31-60 days | 1.58 (1.38-1.80)* | 2.73 (2.37-3.15)* | |
| 61-90 days | 1.90 (1.65-2.19)* | 4.19 (3.59-4.90)* | |
| 91-120 days | 1.74 (1.48-2.06)* | 3.47 (2.93-4.11)* | |
| > 120 days | 1.76 (1.55-2.00)* | 1.99 (1.73-2.28)* | |
| **Marital status** |  |  | |
| Single | 1.01 (0.90-1.14) | 0.99 (0.87-1.14) | |
| Married | **Reference** | | |
| Divorced | 0.68 (0.40-1.16) | 1.28 (0.75-2.17) | |
| Widow | 1.22 (0.81-1.85) | 0.94 (0.61-1.46) | |
| **Nationality** |  |  | |
| Omani | **Reference** | | |
| Non-Omani | 1.31 (0.74-2.34) | 1.07 (0.54-2.13) | |
| **Exempted from all medical service fees  *Statistically significant | | | |

| **Supplementary Table 9: Predictors of missed hospital face-to-face appointments for the Oncology clinic at The Royal Hospital pre and post COVID-19** | | |  |
| --- | --- | --- | --- |
|  | **Pre COVID-19 (N=11704)** | **Post COVID-19**  **(N=9146)** | |
|  | **OR (95% CI)** | **OR (95% CI)** | |
| **Sex** |  |  | |
| Female | **Reference** | | |
| Male | 1.17 (1.02-1.33)* | 1.05 (0.92-1.19) | |
| **Age category** |  |  | |
| 19-30 years old | 1.06 (0.80-1.40) | 1.01 (0.76-1.33) | |
| 31-40 years old | **Reference** | | |
| 41-50 years old | 1.04 (0.86-1.25) | 1.08 (0.91-1.29) | |
| 51-60 years old | 0.98 (0.81-1.19) | 1.12 (0.93-1.34) | |
| 61-70 years old | 0.98 (0.81-1.20) | 1.11 (0.92-1.34) | |
| 71-80 years old | 0.97 (0.77-1.22) | 1.08 (0.86-1.34) | |
| > 80 years old | 1.25 (0.89-1.76) | 0.99 (0.71-1.40) | |
| **Appointment day** |  |  | |
| Sunday | **Reference** | | |
| Monday | 1.09 (0.91-1.31) | 1.10 (0.91-1.33) | |
| Tuesday | 1.01 (0.85-1.19) | 1.34 (1.14-1.58)* | |
| Wednesday | 0.85 (0.71-1.01) | 1.13 (0.95-1.34) | |
| Thursday | 0.87 (0.72-1.05) | 1.35 (1.12-1.62)* | |
| **Service cost** |  |  | |
| Pay visit and registration fees | **Reference** | | |
| Pay all medical service fees | 1.13 (0.62-2.06) | 1.41 (0.69-2.87) | |
| Under social affair coverage** | 0.78 (0.58-1.05) | 1.22 (0.95-1.56) | |
| **Appointment waiting days** |  |  | |
| ≤ 30 days | **Reference** | | |
| 31-60 days | 0.59 (0.49-0.71)* | 1.89 (1.61-2.21)* | |
| 61-90 days | 0.73 (0.60-0.88)* | 1.52 (1.28-1.81)* | |
| 91-120 days | 0.69 (0.59-0.81)* | 1,38 (1.18-1.62)* | |
| > 120 days | 0.89 (0.76-1.04) | 1.48 (1.26-1.74)* | |
| **Marital status** |  |  | |
| Single | 0.93 (0.78-1.11) | 0.99 (0.84-1.16) | |
| Married | **Reference** | | |
| Divorced | 0.92 (0.44-1.94) | 1.16 (0.60-2.24) | |
| Widow | 1.10 (0.72-1.66) | 0.99 (0.65-1.51) | |
| **Nationality** |  |  | |
| Omani | **Reference** | | |
| Non-Omani | 1.12 (0.60-2.08) | 0.88 (0.43-1.84) | |
| **Exempted from all medical service fees  *Statistically significant | | | |

| **Supplementary Table 10: Predictors of missed hospital face-to-face appointments for the Urology clinic at The Royal Hospital pre and post COVID-19** | | |  |
| --- | --- | --- | --- |
|  | **Pre COVID-19 (N=7775)** | **Post COVID-19**  **(N=3845)** | |
|  | **OR (95% CI)** | **OR (95% CI)** | |
| **Sex** |  |  | |
| Female | **Reference** | | |
| Male | 1.26 (1.08-1.47)* | 1.03 (0.86-1.24) | |
| **Age category** |  |  | |
| 19-30 years old | 0.89 (0.70-1.13) | 0.95 (0.71-1.28) | |
| 31-40 years old | **Reference** | | |
| 41-50 years old | 0.99 (0.81-1.23) | 1.04 (0.79-1.36) | |
| 51-60 years old | 0.89 (0.72-1.10) | 1.06 (0.80-1.41) | |
| 61-70 years old | 0.84 (0.69-1.04) | 1.17 (0.90-1.52) | |
| 71-80 years old | 0.79 (0.63-1.00) | 1.05 (0.78-1.42) | |
| > 80 years old | 0.48 (0.33-0.70)* | 1.05 (0.74-1.49) | |
| **Appointment day** |  |  | |
| Sunday | **Reference** | | |
| Monday | No observations recorded for this day | | |
| Tuesday | 0.99 (0.84-1.18) | 0.81 (0.66-1.01) | |
| Wednesday | 0.90 (0.76-1.07) | 1.07 (0.87-1.31) | |
| Thursday | 1.05 (0.89-1.25) | 0.93 (0.75-1.15) | |
| **Service cost** |  |  | |
| Pay visit and registration fees | **Reference** | | |
| Pay all medical service fees | 1.03 (0.36-2.94) | 0.49 (0.13-1.80) | |
| Under social affair coverage** | 1.03 (0.80-1.31) | 1.15 (0.87-1.52) | |
| **Appointment waiting days** |  |  | |
| ≤ 30 days | **Reference** | | |
| 31-60 days | 1.67 (1.09-2.55)* | 3.05 (2.07-4.48)* | |
| 61-90 days | 2.73 (2.00-3.73)* | 3.72 (2.64-5.23)* | |
| 91-120 days | 2.32 (1.70-3.16)* | 4.61 (3.58-5.92)* | |
| > 120 days | 2.65 (1.98-3.56)* | 3.17 (2.62-3.84)* | |
| **Marital status** |  |  | |
| Single | 0.92 (0.78-1.09) | 1.06 (0.86-1.30) | |
| Married | **Reference** | | |
| Divorced | 0.64 (0.23-1.84) | 0.56 (0.06-4.84) | |
| Widow | 0.76 (0.22-2.57) | 0.58 (0.19-1.82) | |
| **Nationality** |  |  | |
| Omani | **Reference** | | |
| Non-Omani | 0.80 (0.27-2.35) | 1.42 (0.38-5.30) | |
| **Exempted from all medical service fees  *Statistically significant | | | |

| **Supplementary Table 11: Predictors of missed hospital face-to-face appointments for the Gastroenterology clinic at The Royal Hospital pre and post COVID-19** | | |  |
| --- | --- | --- | --- |
|  | **Pre COVID-19 (N=4765)** | **Post COVID-19**  **(N=3181)** | |
|  | **OR (95% CI)** | **OR (95% CI)** | |
| **Sex** |  |  | |
| Female | **Reference** | | |
| Male | 1.02 (0.88-1.18) | 1.00 (0.86-1.18) | |
| **Age category** |  |  | |
| 19-30 years old | 1.22 (0.97-1.52) | 1.06 (0.82-1.38) | |
| 31-40 years old | **Reference** | | |
| 41-50 years old | 1.08 (0.89-1.31) | 1.05 (0.85-1.30) | |
| 51-60 years old | 0.94 (0.74-1.18) | 1.08 (0.85-1.38) | |
| 61-70 years old | 1.02 (0.79-1.31) | 0.86 (0.65-1.15) | |
| 71-80 years old | 1.20 (0.84-1.72) | 0.92 (0.58-1.47) | |
| > 80 years old | 1.02 (0.47-2.20) | 1.08 (0.49-2.37) | |
| **Appointment day** |  |  | |
| Sunday | **Reference** | | |
| Monday | 0.54 (0.44-0.66)* | 1.81 (1.43-2.29)* | |
| Tuesday | 0.80 (0.65-0.98)* | 0.89 (0.70-1.15) | |
| Wednesday | 0.48 (0.39-0.60)* | 1.37 (1.06-1.78)* | |
| Thursday | 0.80 (0.64-0.99)* | 2.13 (1.62-2.81)* | |
| **Service cost** |  |  | |
| Pay only visit and registration fees | **Reference** | | |
| Pay all medical service fees | 0.12 (0.02-0.84)* | 0.42 (0.06-3.02) | |
| Under Social Affair coverage** | 1.14 (0.85-1.54) | 0.97 (0.67-1.42) | |
| **Appointment waiting days** |  |  | |
| ≤ 30 days | **Reference** | | |
| 31-60 days | 1.30 (0.94-1.81) | 4.20 (2.88-6.12)* | |
| 61-90 days | 1.65 (1.16-2.34)* | 6.27 (4.28-9.18)* | |
| 91-120 days | 2.53 (1.85-3.46)* | 4.92 (3.49-6.95)* | |
| > 120 days | 2.65 (2.03-3.47)* | 5.57 (4.22-7.36)* | |
| **Marital status** |  |  | |
| Single | 1.11 (0.93-1.32) | 1.04 (0.86-1.26) | |
| Married | **Reference** | | |
| Divorced | 1.41 (0.60-3.28) | 0.69 (0.16-2.95) | |
| Widow | 1.00 (0.39-2.56) | 1.02 (0.29-3.54) | |
| **Nationality** |  |  | |
| Omani | **Reference** | | |
| Non-Omani | 1.00 (0.18-5.64) | 2.11 (0.28-16.04) | |
| **Exempted from all medical service fees  *Statistically significant | | | |

| **Supplementary Table 12: Characteristics of virtual clinic appointments for each unique patient for all appointments stratified by attended and missed appointments** | | | | | | |
| --- | --- | --- | --- | --- | --- | --- |
|  | **Characteristics of Patients (N)** | | **Characteristics of Appointments (N)** | | **Attended**  **N (%)** | **Missed**  **N (%)** |
|  | **11437** | | **23100** | | **15653 (67.8)** | **7447 (32.2)** |
| **Appointment month** *p value<0.001 | | | | | | |
| January | 1222 (15.7) | 2453 (10.6) | | 1729 (70.5) | | 724 (29.5) |
| February | 1345 (11.8) | 2581 (11.2) | | 1793 (69.5) | | 788 (30.5) |
| March | 1801 (15.7) | 3173 (13.7) | | 2195 (69.2) | | 978 (30.8) |
| April | 45 (0.4) | 87 (0.4) | | 72 (82.8) | | 15 (17.2) |
| May | 238 (2.1) | 364 (1.6) | | 284 (78.1) | | 80 (21.9) |
| June | 1153 (10.1) | 2293 (9.9) | | 1611 (70.3) | | 682 (29.7) |
| July | 853 (7.5) | 1812 (7.8) | | 1273 (70.3) | | 539 (29.7) |
| August | 637 (5.6) | 1482 (6.4) | | 917 (61.9) | | 565 (38.1) |
| September | 950 (8.3) | 2106 (9.1) | | 1314 (62.4) | | 792 (37.6) |
| October | 1055 (9.2) | 2114 (9.2) | | 1424 (67.4) | | 690 (32.6) |
| November | 930 (8.1) | 2045 (8.9) | | 1256 (61.4) | | 789 (38.6) |
| December | 1208 (10.6) | 2590 (11.2) | | 1785 (69.0) | | 805 (31.0) |
| **Marital status** *p value<0.001 | | | | | | |
| Child | 2053 (18.0) | 3548 (15.4) | | 2744 (77.3) | | 804 (22.7) |
| Single | 2974 (26.0) | 6366 (27.6) | | 4230 (66.4) | | 2136 (33.6) |
| Married | 6260 (54.7) | 12940 (56.0) | | 8502 (65.7) | | 4438 (34.3) |
| Divorced | 60(0.5) | 106 (0.5) | | 70 (66.0) | | 36 (34.0) |
| Widow | 90 (0.8) | 140 (0.6) | | 107 (76.4) | | 33 (23.6) |
| **Appointment year** *p value<0.001 | | | | | | |
| 2020 | 7069 (61.8) | 14893 (64.5) | | 9936 (66.7) | | 4957 (33.3) |
| 2021 | 4368 (38.2) | 8207 (35.5) | | 5717 (69.6) | | 2490 (30.4) |
| **Nationality** *p value<0.001 | | | | | | |
| Omani | 110941 (95.7) | 22218 (96.2) | | 15151 (68.2) | | 7067 (31.8) |
| Non Omani | 496 (4.3) | 882 (3.8) | | 502 (56.9) | | 380 (43.1) |
| *p value calculated using the Tableone R package which summarises categorical and continuous variables. The chi-squared test was used to calculate the p value for categorical variable and one-way analysis of variance (ANOVA) for continuous variables. | | | | | | |

| **Supplementary Table 13: Distribution of the virtual clinic appointments scheduled within different medical speciality at The Royal Hospital stratified by attended and missed appointments** | | | | | |
| --- | --- | --- | --- | --- | --- |
|  | **Characteristics of Patients (N)** | **Characteristics of Appointments (N)** | **Attended**  **N (%)** | | **Missed**  **N (%)** |
|  | **11437** | **23100** | **15653 (67.8)** | | **7447 (32.2)** |
| **Medical speciality**  *p value<0.001 | | | | | |
| Adolescent Medicine | 47 (0.4) | 139 (0.6) | 289 (67.2) | 141 (32.8) | |
| Anaesthesia | 373 (3.3) | 631 (2.7) | 444 (70.0) | 190 (30.0) | |
| Cardiology | 449 (3.9) | 647 (2.8) | 478 (72.1) | 185 (27.9) | |
| Chest Medicine | 358 (3.1) | 597 (2.6) | 400 (65.1) | 214 (34.9) | |
| Clinical Haematology | 347 (3.0) | 392 (1.7) | 389 (96.3) | 15 (3.7) | |
| Clinical Immunology and Allergy | 136 (1.2) | 173 (0.7) | 10 (5.5) | 173 (94.5) | |
| Diabetes and Endocrine | 12131 (0.6) | 5684( 24.6) | 2845 (50.2) | 2818 (49.8) | |
| Gastroenterology | 668 (5.8) | 834 (3.6) | 665 (78.3) | 184 (21.7) | |
| Genetics Disorders | 56 (0.5) | 112 (0.5) | 559 (78.3) | 155 (21.7) | |
| Infectious Diseases | 175 (1.5) | 319 (1.4) | 214 (67.1) | 105 (32.9) | |
| Medical: Endo-Metabolic | 377 (3.3) | 824 (3.6) | 675 (79.4) | 175 (20.6) | |
| Nephrology | 408 (3.6) | 940 (4.1) | 848 (87.1) | 126 (12.9) | |
| Obstetrics and Gynaecology | 847 (7.4) | 1287 (5.6) | 921 (71.6) | 366 (28.4) | |
| Oncology | 162 (1.4) | 274 (1.2) | 185 (66.1) | 95 (33.9) | |
| Paediatric | 2867 (25.0) | 5381(23.3) | 3139 (73.3) | 1146 (26.7) | |
| Rheumatology | 1640 (14.3) | 2948 (12.8) | 2227 (74.3) | 769 (25.7) | |
| Staff Clinic | 156 (1.4) | 186 (0.8) | 7 (3.8) | 179 (96.2) | |
| Surgery | 719 (6.3) | 1228 (5.3) | 910 (72.6) | 344 (27.4) | |
| Urology | 439 (3.8) | 504 (2.2) | 443 (86.0) | 72 (14.0) | |
| *p value calculated using the Tableone R package which summarises categorical and continuous variables. The chi-squared test was used to calculate the p value for categorical variable and one-way analysis of variance (ANOVA) for continuous variables. † The Cooperation Council for the Arab States of the Gulf | | | | | |

| **Supplementary Table 14: Effects of distance between patient’s residence and Royal Hospital on missed appointments post COVID-19 face to-face and post COVID-19 virtual clinics** | | | | |
| --- | --- | --- | --- | --- |
| **Governorate** | **Minimum-Maximum Distance to The Royal Hospital (Km)** | **Odd Ratio (95% CI)** | | |
|  |  | **Post**  **COVID-19**  **Face-to-Face** | **Post**  **COVID-19**  **Virtual** | **^ Interaction** |
| Muscat | 1-90 | **Reference** | | |
| South Batina | 50-165 | 1.37 (1.16-1.61)* | 0.69 (0.61-0.78)* | 1.09 (0.99-1.19) |
| AL Dhakiliya | 70-206 | 1.50 (1.18-1.90)* | 0.83 (0.73-0.93)* | 1.07 (0.96-1.18) |
| North Batina | 114-262 | 1.07 (0.92-1.24) | 0.84 (0.73-0.96)* | 0.98 (0.89-1.08) |
| North Sharqiya | 173-290 | 1.01 (0.95-1.08) | 0.83 (0.71-0.97)* | 0.96 (0.91-1.01) |
| South Sharqiya | 205-320 | 1.06 (1.00-1.12)* | 0.71 (0.61-0.83)* | 0.98 (0.92-1.03) |
| AL Dhahira | 245-340 | 0.98 (0.92-1.04) | 0.78 (0.63-0.98)* | 0.96 (0.91-1.02) |
| AL Buriami | 285-350 | 1.21 (1.13-1.30)* | 0.98 (0.70-1.37) | 1.17 (1.09-1.26)* |
| AL Wusta | 365-490 | 1.08 (1.00-1.16)* | 0.40 (0.19-0.84)* | 1.06 (0.98-1.14) |
| Musandam | 540-620 | 1.03 (0.94-1.12) | 0.79 (0.46-1.35) | 0.92 (0.85-1.00) |
| Dhofar | 800-1120 | 1.01 (0.75-1.35) | 0.62 (0.41-0.93)* | 0.82 (0.66-1.02) |
| ^ Interaction: models adjusted with an interaction term for Post COVId-19 Face-to-Face and Post COVID-19 Virtual appointments.  *Statistically significant | | | | |
